# Comprehensive analysis of the metabolic and genomic features of tannin-transforming *Lactiplantibacillus plantarum* strains

**DOI:** 10.1038/s41598-022-26005-4

**Published:** 2022-12-27

**Authors:** Elena C. Pulido-Mateos, Jacob Lessard-Lord, Denis Guyonnet, Yves Desjardins, Denis Roy

**Affiliations:** 1 Institut sur la Nutrition et les Aliments Fonctionnels de l’Université Laval, Faculté des Sciences de l’agriculture et de l’alimentation, Université Laval, Quebec, QC Canada; 2 Laboratoire de Génomique Microbienne, Département des Sciences des Aliments, Faculté des Sciences de l’agriculture et de l’alimentation, Université Laval, Quebec, QC Canada; 3 Symrise Taste, Nutrition and Health, Rennes, France

**Keywords:** Applied microbiology, Bacterial genes, Bacterial genetics

## Abstract

Extracellular tannase *Lactiplantibacillus plantarum*-producing strains (TanA+) release bioactive metabolites from dietary tannins. However, there is a paucity of knowledge of TanA+ strains and their hydrolyzing capacities. This study aimed to shed light on the metabolic and genomic features of TanA+ *L. plantarum* strains and to develop a screening technique. The established spectrophotometric was validated by UPLC-UV-QToF. Eight of 115 screened strains harbored the *tanA* gene, and six presented TanA activity (PROBI S126, PROBI S204, RKG 1-473, RKG 1-500, RKG 2-219, and RKG 2-690). When cultured with tannic acid (a gallotannin), TanA+ strains released 3.2−11 times more gallic acid than a lacking strain (WCFS1) (*p* < 0.05). TanA+ strains with gallate decarboxylase (n = 5) transformed this latter metabolite, producing 2.2–4.8 times more pyrogallol than the TanA lacking strain (*p* < 0.05). However, TanA+ strains could not transform punicalagin (an ellagitannin). Genomic analysis revealed high similarity between TanA+ strains, as only two variable regions of phage and polysaccharide synthesis were distinguished. A phylogenetic analysis of 149 additional genome sequences showed that *tanA *harboring strains form a cluster and present two bacteriocin coding sequences profile. In conclusion, TanA+ *L. plantarum* strains are closely related and possess the ability to resist and transform gallotannins. TanA can be screened by the method proposed herein.

## Introduction

Tannins are a diverse group of high molecular weight polyphenols present in several fruits and nuts, such as mango, pomegranate, and almonds. Tannin intake has been associated with anti-obesity, antidiabetic, cardioprotective, and anticancer effects^1^. Yet, the extent of these benefits appears to be strongly influenced by the conversion of these polyphenols polymers into more bioaccessible phenolic metabolites by the gut microbiota. For example, mango gallotannins (the simplest tannin type) were more effective in reducing an atherosclerosis risk biomarker (plasminogen activator inhibitor-1) and a hyperglycemia biomarker (hemoglobin A1c) when they were converted into 4-*O*-methylgallic acid, a conjugated microbial-derived metabolite^2^. However, certain individuals lack the microbial species that transform these phenolic compounds. For instance, subjects with obesity display a reduced intestinal tannase activity, a key bacterial enzyme involved in gallotannin metabolism^3^. Indeed, compared to individuals with obesity, lean ones produce higher quantities of gallotannin metabolites, showing up to three times more 4-*O*-methyl-gallic acid (a microbial-derived gallotannin metabolite) in their blood plasma^3^. Strategies to increase the production of tannin-derived microbial metabolites are thus sought, particularly for individuals lacking the bacterial species involved in tannin metabolism.

A promising avenue to enhance the release of bioactive tannin metabolites is using probiotic bacteria with suitable metabolizing capacity. To this effect, *Lactiplantibacillus plantarum* is a bacterium of interest because of its wide repertoire of tannases and other (poly)phenol-associated enzymes (PAZymes). Indeed, the *L. plantarum* genome encodes three tannases: a widely-spread intracellular tannase (TanB), a strain-specific intracellular broad esterase (Est_1092), and an extracellular tannase (TanA), the latter only described in the ATCC 14917 strain^4,5^. Noteworthy, only TanA can hydrolyze gallotannins, as these high molecular weight molecules cannot enter the microbial cell^5^. The product of this hydrolysis, gallic acid, can be further transformed by the gallate decarboxylase enzyme into pyrogallol, a molecule with anticarcinogenic and anti-obesogenic properties^6–8^. Hence, *L. plantarum* strains with these enzymatic capacities may be good candidates to enhance gallotannin-metabolite-production in the gut or through food fermentation. However, because *tanA* (encoding TanA enzyme) was reported as a non-inducible gene^5^, it is unclear if its enzymatic activity induces a valuable production of gallotannin metabolites (gallic acid and pyrogallol). In which case, the detoxifying action of the TanA might provide the producing strains an ability to adjust their metabolism to thrive in tannin-rich niches (improved fitness)^9^. Another interrogative is if TanA producing strains (TanA+) hydrolyze ellagitannins, another type of tannins displaying variable and complex structures with esterified hexahydroxydiphenoyl moieties^10^. Indeed, ellagitannins have unique molecular configurations made by galloyl units strongly linked by C–C bonds^1^, which might result in molecules harder to hydrolyze.

Among lactic acid bacteria, the *L. plantarum* species attracts attention for its long genome (2.9–3.7 Mb) and high genetic diversity^11^. Some of the most variable genes confer a probiotic potential to the strains, especially those involved in the synthesis of exopolysaccharides, capsular polysaccharides, and bacteriocins^12^. In an effort to understand the genetic distribution of the strains, Choi et al*.* (2021) phylogenetically characterized fifty-four complete genome sequences and found that the *L. plantarum* subsp. *plantarum* species can be split into three lineages (A, B, and C), each displaying a different bacteriocin gene profile^13^. Since the presence of the *tanA* gene in *L. plantarum* is highly strain-specific, it is possible that *tanA* harboring strains belong to a specific lineage and share functional gene characteristics.

This study aimed to assess the metabolic and genomic characteristics of TanA+ *L. plantarum* strains and to establish an efficient and rapid screening technique for their identification. The capacity of the selected strains to metabolize gallic acid, as well as tannic acid (gallotannin) and punicalagin (ellagitannin), was evaluated. Moreover, the genotype and fitness of the TanA+ strains were studied and related to the metabolic abilities presented by the strains. Finally, we analyzed the phylogenetic relationship between *tanA*harboring strains and their bacteriocin coding sequence profile.

## Methods

### Strains and culture conditions

The *L. plantarum* ATCC 14917 and *L. plantarum* WCFS1 strains were purchased from the American Type Culture Collection. For the screening, thirty-eight *L. plantarum* strains isolated from human and plant sources were provided by the Probi company (Probi AB, Lund, Sweden). In addition, seventy-seven *L. plantarum* isolates collected from bovine raw milk and forages (hay and silage)^14^ were included in the study. The identity of the *L. plantarum* isolates was confirmed by the PCR assay described by Torriani and Dellaglio^15^. A stock culture of each isolate was stored at − 80 °C in Man-Rogosa-Sharpe (MRS) medium supplemented with 20% of glycerol. For subsequent experiments, strains were reactivated in MRS medium, and the third sub-culture was taken for inoculation.

The basal medium developed by Rozès and Peres^16^ was chosen to evaluate the performance of the strains in the presence of (poly)phenols with some modifications (RP-M). Glucose was exchanged for galactose to avoid a possible carbon catabolite repression^17^. This medium has been used to demonstrate the catabolic activity of *L. plantarum* ATCC 14917 towards tannic acid^5^. RP-M medium was also supplemented with 1% of DMSO to facilitate the dissolution of (poly)phenols and 1.9% of β-glycerophosphate disodium salt hydrate to enhance its buffering capacity. The pH of RP-M medium was adjusted to 5.0 to prevent tannin degradation at higher pH values. The medium was sterilized by filtration.

### Comparison of released gallotannin-metabolites of a TanA+ versus a TanA lacking strain

The released gallotannin-metabolites of a TanA+ (ATCC 14917) and a TanA lacking strain (WCFS1) were quantified throughout their growth in RP-M medium supplemented with tannic acid (50 µM) (Alfa Aesar, Ward Hill, MA, USA). Briefly, tubes containing the supplemented medium were inoculated with 1% of a 13 h-MRS culture of each strain. To analyze gallic acid and pyrogallol metabolites, 650 µL-supernatant samples were taken at the beginning of the fermentation and after days 1, 2, 5, 7, and 10.

### Development of a visual method to detect TanA+ *L. plantarum strains*

To identify TanA+ strains, we adapted the method developed by Sharma et al.^18^, which detects extracellular tannase-producing fungus. This method is based on the reaction of rhodanine with the two vicinal hydroxyl groups of gallic acid, which forms a red complex^19^. First off, RP-M medium supplemented with tannic acid (50 µM) was inoculated at 1% with a 13-h MRS culture of either ATCC 14917 (TanA+) or WCFS1 (TanA lacking) control strains and incubated at 30 °C without agitation. Samples (1 mL) were taken at 0, 2, 5, 7, and 10 days of incubation, and culture supernatants were obtained by centrifuging 10,000×*g* for 10 min at 20 °C. To induce the reaction, 150 µl of rhodanine (0.667% in methanol w/v) were added to 250 µl of supernatant, followed by 100 µl of potassium hydroxide (0.5 N). The reaction mix was incubated for 5 min at 30° C and then diluted with 1 mL of distilled water. A strong pink coloration indicated TanA activity, while a pale pink tone indicated its absence (Fig. S1a, supplementary material). To estimate TanA activity, a calibration curve was calculated from 10 to 400 µM of gallic acid (3,4,5-Trihydroxybenzoic acid) (Alfa Aesar, Ward Hill, MA, USA) solutions diluted in RP-M medium, and the absorbance was read at 520 OD (Fig. S1b, supplementary material). The non-inoculated culture medium, exposed to the same incubation conditions, was used as blank. The method was validated by correlating the spectrophotometric results obtained with the TanA+ strain culture samples at different fermentation times with the results obtained by UPLC-UV-QToF.

### Screening of TanA+ *L. plantarum strains*

To screen *tanA* harboring strains, the chromosomal DNA of 115 *L. plantarum* isolates was obtained as described by Gagnon et al.^14^. For the PCR assay, the primers proposed by Jimenez et al.^5^, targeting the *tanA* gene in *L. plantarum,* were used. The amplification products were visualized in 1% (w/v) agarose gels after electrophoresis.

**Figure 1:**
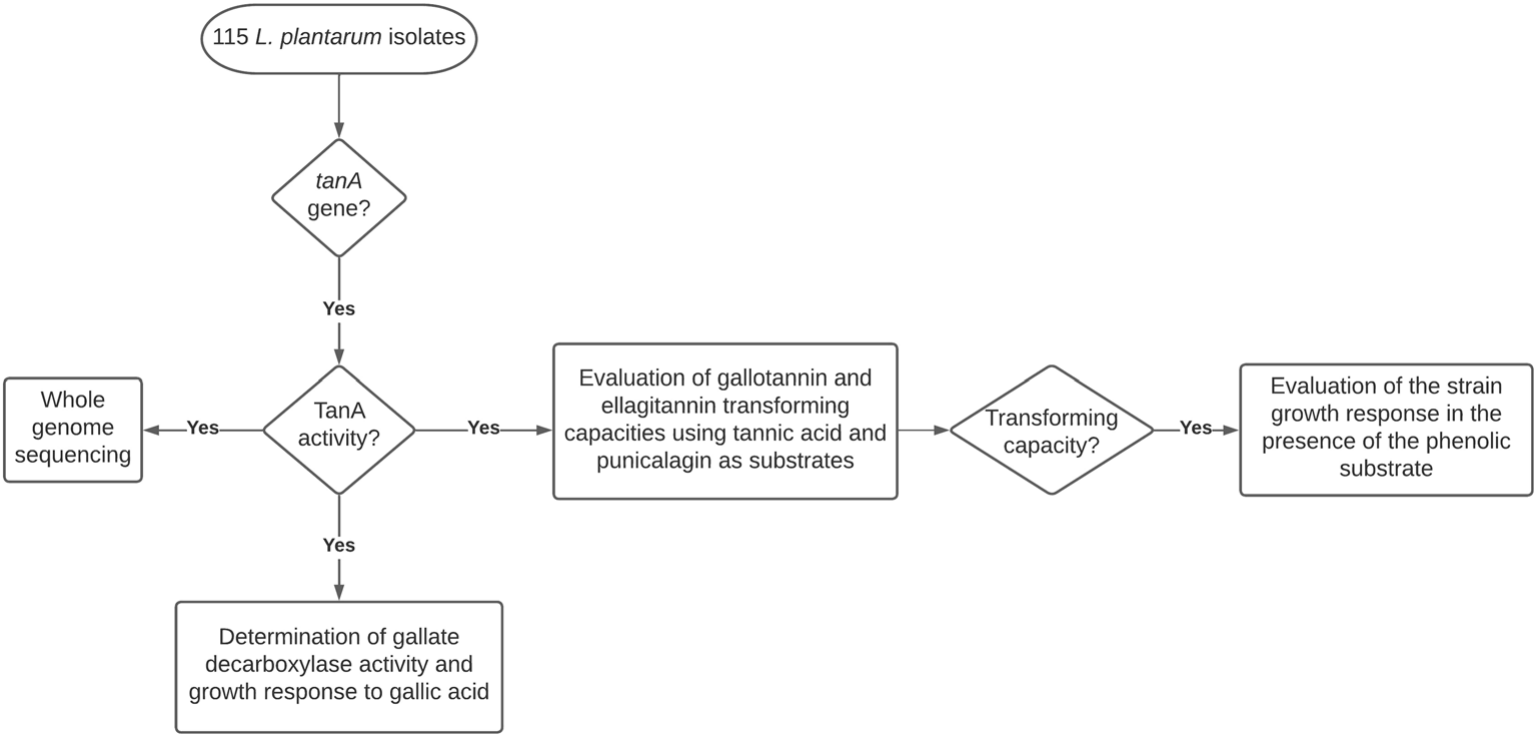
Flow diagram of *L. plantarum* screening, sequencing, and the evaluation of the strain metabolic activities and growth responses in the presence of (poly)phenols.

The *tanA* harboring strains were further screened for TanA activity (Fig. 1) as previously described.

### Transforming capacities of the selected strains towards (poly)phenols

The capacity of TanA+ strains to transform gallic acid, tannic acid, and punicalagin (Sigma-Aldrich, USA) was tested by cultivating the strains in RP-M medium supplemented with each phenolic compound. The gallic acid dose (500 µM) was nearly equivalent to that of tannic acid (50 µM), supposing that each hydrolyzed mol of tannic acid liberates ~ 10 mols of this phenolic acid. Tannic acid and punicalagin concentrations were chosen to allow the quantification of the compounds and their metabolites above the detection limits of the analytical methods. These doses might be physiologically obtained with the intake of ~ two cups of mango or pomegranate, respectively, assuming a 4-L gut volume^20^. The WCFS1 strain was used as a positive control for gallate decarboxylase activity and as a TanA lacking control, while the ATCC 14917 strain was used as TanA+ control. The non-inoculated medium exposed to the same incubation conditions was also used as a negative control for gallic acid, tannic acid, and punicalagin transformation. To perform the test, the (poly)phenol-supplemented media were inoculated with 1% of 13-h MRS culture of each strain and incubated at 30 °C without agitation. At the end of the fermentation (10 d), the supernatants of each strain were collected and analyzed*.* Additionally, to detect any change throughout the fermentation with punicalagin, culture supernatants of the TanA+ (ATCC 14917) and the TanA lacking (WCFS1) reference strains were taken after 0, 1, 2, 5, and 7 days of incubation.

### Chemical analysis of phenolic compounds by ultra-performance liquid chromatography–ultraviolet–quadrupole-time of flight (UPLC–UV–QToF)

A Waters ACQUITY® I-Class UPLC coupled to a Synapt G2-Si was used to analyze gallic acid, pyrogallol, and punicalagin. A volume of 5 µL was injected onto an ACQUITY® UPLC HSS T3 (2.1 × 100 mm, 1.8 µm) column protected with a VanGuard pre-column (2.1 × 5 mm, 1.8 µm) and heated to 30 °C. A binary gradient of water (mobile phase A) and acetonitrile (mobile phase B), both acidified with 0.1% formic acid, was used for the elution with a flow rate of 0.300 mL/min. Specific gradient and sample preparation are described below. Synapt G2-Si QToF was operated in negative electrospray ionization in resolution mode (resolution ≈ 25 000). Data were acquired in MS^E^ mode, with a scanning range of 50 to 2000 (m/z), with a collision energy ramp of 20–50 V applied in the transfer cell in the high energy function. Source parameters were as follows: capillary voltage of 2.4 kV, sampling cone of 40 V, source offset of 80 V, source temperature of 120 °C, desolvation temperature of 250 °C, cone gas flow of 50 L/h, desolvation gas flow of 600 L/h. Leucine-enkephalin was infused at a flow rate of 10 µL/min and was used as an internal mass calibrant. Quantification was done using UV signal (266 nm for pyrogallol, 272 nm for gallic acid, and 360 nm for punicalagin), and compound identification was confirmed using MS signal.

For the pyrogallol and gallic acid quantification, samples were diluted by 2 in water with 0.1% of formic acid and passed through a 0.22 µm Nylon filter. The gradient was as follows: 1% of mobile phase B at 0 min, 1% at 2 min, 61% at 10 min, 95% at 13 min, 1% at 13.1 min, and 1% at 16 min. For the punicalagin, ellagic acid, and urolithins quantifications, samples were diluted by 3 in methanol acidified with 0.5% of formic acid and passed through a 0.22 µm Nylon filter. Using the gradient proposed by García-Villalba et al.^21^: 0 min, 5% of mobile phase B; 18% at 7 min, 28% at 17 min, 50% at 22 min, 90% at 27 min, 90% at 28 min, 5% at 29 min, and 5% at 33 min.

### Determination of the growth rate of the TanA+ strains in the presence of (poly)phenols

Growth was measured by reading optical density (OD) using a Powerwave XS2 microplate reader (Biotek). Each microplate well was filled with 200 µl of RP-M medium, with or without (poly)phenols, inoculated with 1% of a 13-h MRS culture. The reader program was set to incubate each plate at 30 °C for 48 h and collect the OD data every 30 min. Uninoculated wells filled with medium were used as blank. Each independent experiment was performed on a different plate in triplicate. Maximum specific growth rates (µ_max_) were obtained by calculating the slope of the natural logarithm (ln[OD_600_]) versus time, using a sliding window of four points. Growth rates registered with the non-supplemented medium were used to normalize the data of each strain. The percentage of µ_max_ variation was calculated the Eq. (1).

1 $$\% \,\,{\text{of}}\,\,\mu {\text{max}}\,\,{\text{variation}} = \frac{{\mu {\text{max}}\,\,{\text{obtained}}\,\,{\text{with}}\,\,{\text{the}}\,\,{\text{(poly)phenol}}\,\,{\text{supplemented}}\,\,{\text{medium}} \cdot 100}}{{\mu {\text{max}}\,\,{\text{obtained}}\,\,{\text{with}}\,\,{\text{the}}\,\,{\text{non - supplemented}}\,\,{\text{medium}}}}$$

TanA+ strains growth was evaluated in the presence of tannic acid (5 µM, 25 µM, and 50 µM) and equivalent gallic acid concentrations (50 µM, 250 µM, and 500 µM). A non-transforming strain was included in each test to determine if the metabolic activity provides them an advantage to grow in the presence of these (poly)phenols. The TanA lacking strain, WCFS1, and the gallate decarboxylase lacking strain, RKG 1-500, were used for this matter.

### Genomic analysis of the TanA+ selected strains

The chromosomal DNA of the six selected strains was extracted as previously described and sent for sequencing to the Institute of Integrative Biology and Systems (Université Laval, https://www.ibis.ulaval.ca/). Sequencing was performed using the Illumina MiSeq platform, able to generate 300 bp paired-end reads. Raw reads were assembled using Uniclycler^22^ in the Bacterial and Viral Bioinformatics Resource Center (BV-BRC, https://www.bv-brc.org/) (formerly, PATRIC)^23^. Genome scaffolding was completed using M_E_D_U_S_A_ scaffolder^24^, with *L. plantarum* WCFS1 genome as the reference. Genome functions were annotated with the RAST tool kit^25^ in the BV-BRC.

### Comparative genomic analysis of the TanA+ selected strains

The genetic features of the selected strains were compared using two strains’ genomes as reference (WCFS1 and ATCC 14917), employing the proteome comparison tool available in the BV-BRC. In addition, multiple alignments of DNA and amino acid sequences of the tannin-transforming enzymes were performed in the BV-BRC.

### Safety assessment of TanA+ selected strains based on their genome

To know if the selected TanA+ strains are potentially safe, two main safety aspects of probiotic bacteria were evaluated: the transferability of antibiotic resistance genes of the strains and their capacity to produce biogenic amines. Antibiotic resistance and biogenic amine production genes were identified with the KEGG database^26–28^ (release 102.0) using BlastKOALA. Antimicrobial resistance genes were inspected in Brite- > ko01504 and the biogenic amine production genes were manually checked by searching the KEGG IDs suggested by Chokesajjawatee et al.^29^. The presence of mobile elements in *L. plantarum* genomes was determined to study the potential transferability of the antimicrobial resistance genes. Prophages were predicted using the PHASTER (http://phaster.ca/)^30^ and putative plasmidic contigs with plasmidSPAdes^31^. In addition, chromosomal antimicrobial resistance genes were checked for flanking transposable elements in the region viewer of the BV-BRC.

### Phylogenetic analysis and characterization of the tanA harboring strains

A phylogenetic tree was constructed from 149 complete and good-quality available genomes of *L. plantarum* in the BV-BRC database (Table S1, supplementary material), along with the ones of the six selected strains. *TanA* gene was searched across the 149 *L. plantarum* genomes with the BLAST tool. For the dendrogram, 1000 single-copy core genes were aligned with MUSCLE^32^, and a maximum-likelihood tree was built using the BV-BRC tool^33^. The image of the tree was obtained with the Interactive Tree Of Life tool (https://itol.embl.de/). A second tree, with the resulting *tanA* harboring strains and the selected TanA+ strains was performed using the same approach.

*L. plantarum* strains harboring the *tanA* gene and the selected TanA+ strains were characterized according to their bacteriocin gene profile using BAGEL4^34^ as previously described^13^. In addition, the gallate decarboxylase genomic features (LpdB, LpdC) were determined in these sequences using BLAST and the amino acid sequences of the WCFS1 strain as queries in the BV-BRC.

### Statistical analysis

The results are expressed as the mean ± standard error of the mean (SEM) of three independent experiments unless stated otherwise. For statistical inferences, models were evaluated for normality by the Shapiro–Wilk test and the visual inspection of the distribution of residuals in Quantile–Quantile plots. Statistical analyses were performed in GraphPad Prism 9.3.1. Differences were considered significant at *p* < 0.05.

## Results

### Gallotannin metabolite production by *L. plantarum* reference strains with and without TanA activity

**Figure 2:**
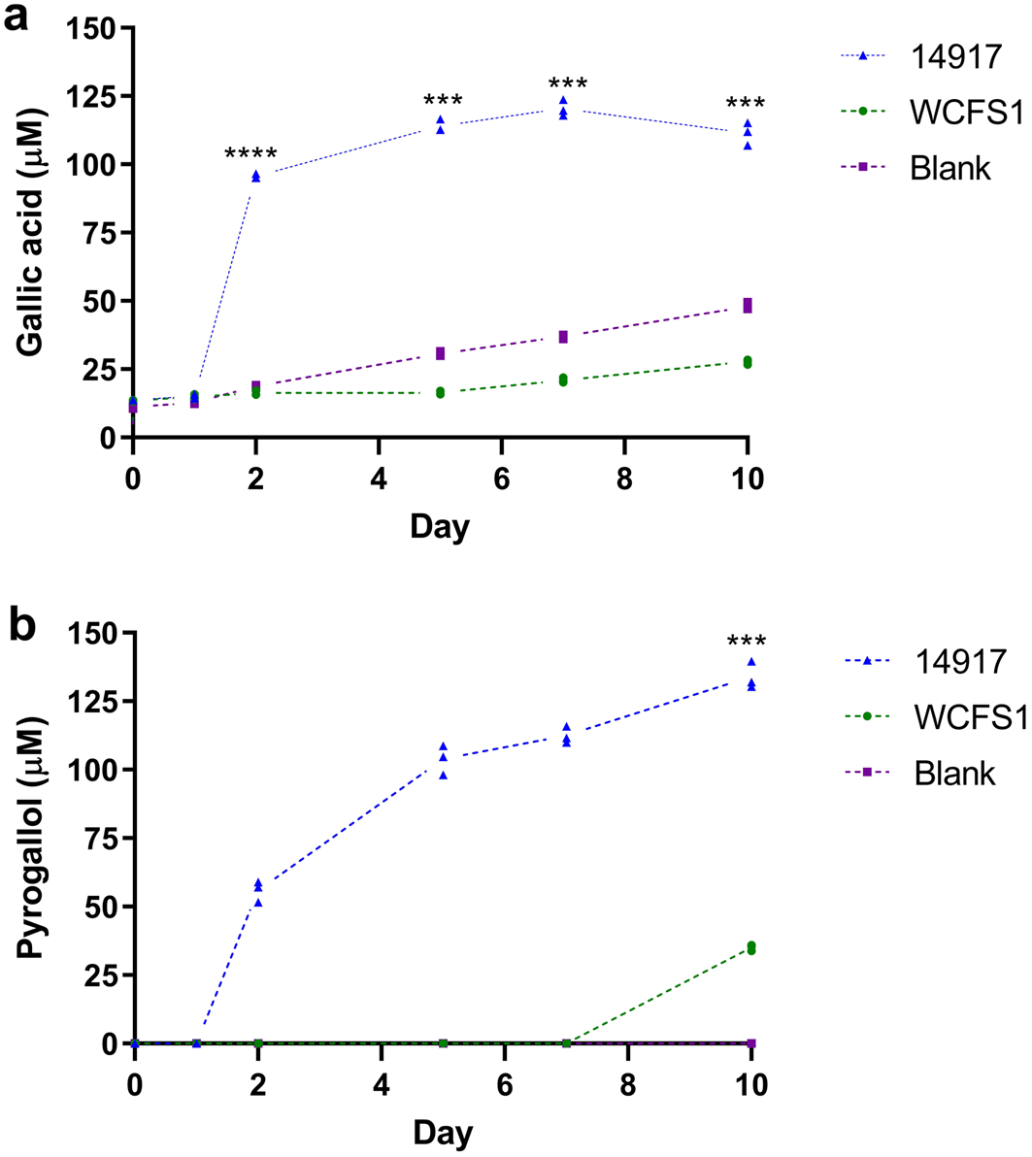
Time course phenolic metabolite-release by a TanA_+_ and a TanA lacking *L. plantarum* strain cultured in a tannic acid-rich medium. (**a**) Gallic acid; (**b**) pyrogallol. Blue triangle, TanA+ ATCC 14917 strain; green circle, TanA lacking strain, WCFS1; purple square, blank (uninoculated media). Values of three independent experiments are shown. A two-way repeated-measures ANOVA with the Geisser–Greenhouse correction followed by Dunnett post hoc test was used to compare groups each time, using WCFS1 strain as a control. ***p ≤ 0.001, ****p ≤ 0.0001. A residual gallic acid from the tannic acid reagent can be observed at time zero. Due to tannic acid degradation at incubation conditions (30° C), gallic acid increases over time.

First, we determined if a TanA+ reference strain (*L. plantarum* ATCC 14917) could release more gallotannin-metabolites (from tannic acid) than a TanA lacking strain (*L. plantarum* WCFS1). Overall, *L. plantarum* ATCC 14917 released 7.5 times more gallic acid than the WCFS1 strain (106.7 ± 2.9 vs. 14.2 ± 0.9 µmols, *p* < 0.001), mainly at the beginning of its stationary growth phase (Fig. 2a) that starts after 20 h (Fig. S2, supplementary material). This higher gallic acid availability allowed ATCC 14917 strain to release 3.8 times more pyrogallol than the TanA lacking strain (134 ± 4.9 vs. 35 ± 1.1 µmols, *p* < 0.001) since this latter could only transform the residual gallic acid of the reagent or the one spontaneously released throughout the fermentation (Fig. 2a). *L. plantarum* ATCC 14917 steadily produced pyrogallol throughout its stationary growth phase (Fig. 2b).

### Development of a screening method to detect and quantify TanA activity in *L. plantarum*

**Figure 3:**
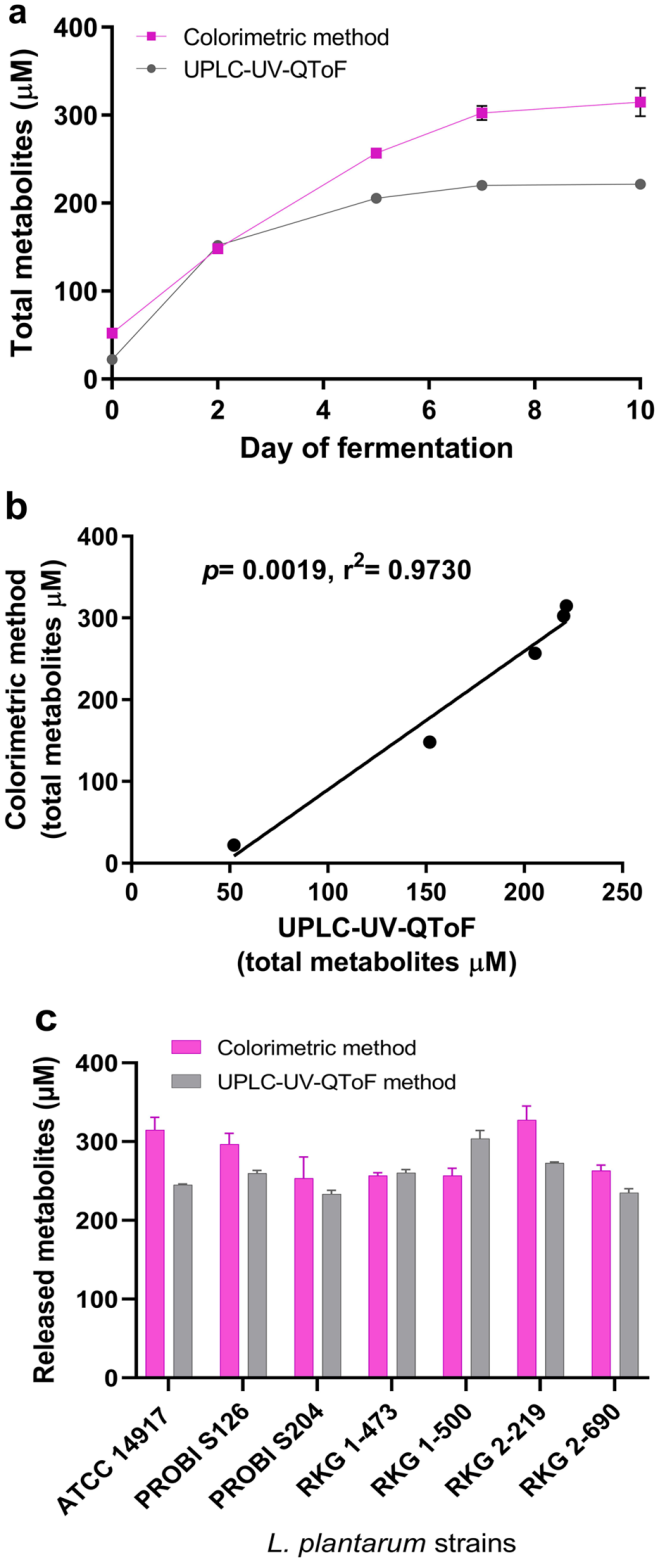
Spectrophotometric method to screen TanA activity in *L. plantarum*. (**a**) Total metabolites (gallic acid + pyrogallol) released by a TanA+ strain (ATCC 14917) throughout a tannic acid-rich medium fermentation. In pink, the results obtained by the proposed colorimetric method; in gray, the ones obtained by UPLC-UV-QToF. (**b**) Pearson correlation between the colorimetric and the UPLC-UV-QToF method. (**c**) Total released metabolites by the six selected *L. plantarum* strains after 10 d of fermentation. In pink, the results of the colorimetric method; in gray, the ones of UPLC-UV-QToF.

A practical screening method to identify TanA+ strains was developed, as detailed above. TanA+ strains are visually distinguished by a strong pink coloration, while TanA lacking strains present a much lower tonality (Fig. S1a, supplementary material). Moreover, the absorbance obtained by this method was well correlated with the results obtained by UPLC-UV-QToF (Pearson correlation, *p* = 0.0019, r^2^ = 0.9730) (Fig. 3), indicating that this technique may help to quantify the released metabolites resulting from TanA activity. However, because both metabolites (i.e., gallic acid and pyrogallol) react simultaneously with the rhodanine reagent^35^, the estimate is not as accurate as when these metabolites are quantified separately by UPLC-UV-QToF (Fig. 3a,c).

### Screening of TanA+ strains and estimation of total released metabolites

To select TanA+ strains candidates, 115 *L. plantarum* isolates were first screened by PCR for the presence of the *tanA* gene, encoding the extracellular tannase (Table S2, supplementary material). The *tanA* gene was detected in eight isolates: *L. plantarum* RKG 1-500 and *L. plantarum* RKG 2-439, isolated from milk; *L. plantarum* RKG 1-473, *L. plantarum* RKG 1-611, and *L. plantarum* RKG 2-219, isolated from corn silage; *L. plantarum* PROBI S126 and *L. plantarum* PROBI S204, isolated from infant feces, and *L. plantarum* RKG 2-690, isolated from grass silage. Among the eight selected *tanA* harboring strains, TanA activity was positive in six, while two lacked this enzymatic capacity (*L. plantarum* RKG 1-611 and *L. plantarum* RKG 2-439) (Table S2, supplementary material).

### Metabolic capacities of the selected TanA+ strains

#### Transforming capacities of TanA+ strains towards gallic acid

The gallate decarboxylase activity of TanA+ strains was evaluated by measuring their capacity to transform the gallic acid supplemented in their medium into pyrogallol (quantified by UPLC-UV-QToF). Except for *L. plantarum* RKG 1-500, all selected strains exhibited gallate decarboxylase activity. At the end of the fermentation, all *L. plantarum* strains with gallate decarboxylase activity had transformed the totality of the gallic acid (Fig. S3, supplementary material).

#### Transforming capacities of TanA+ strains towards tannic acid

**Figure 4:**
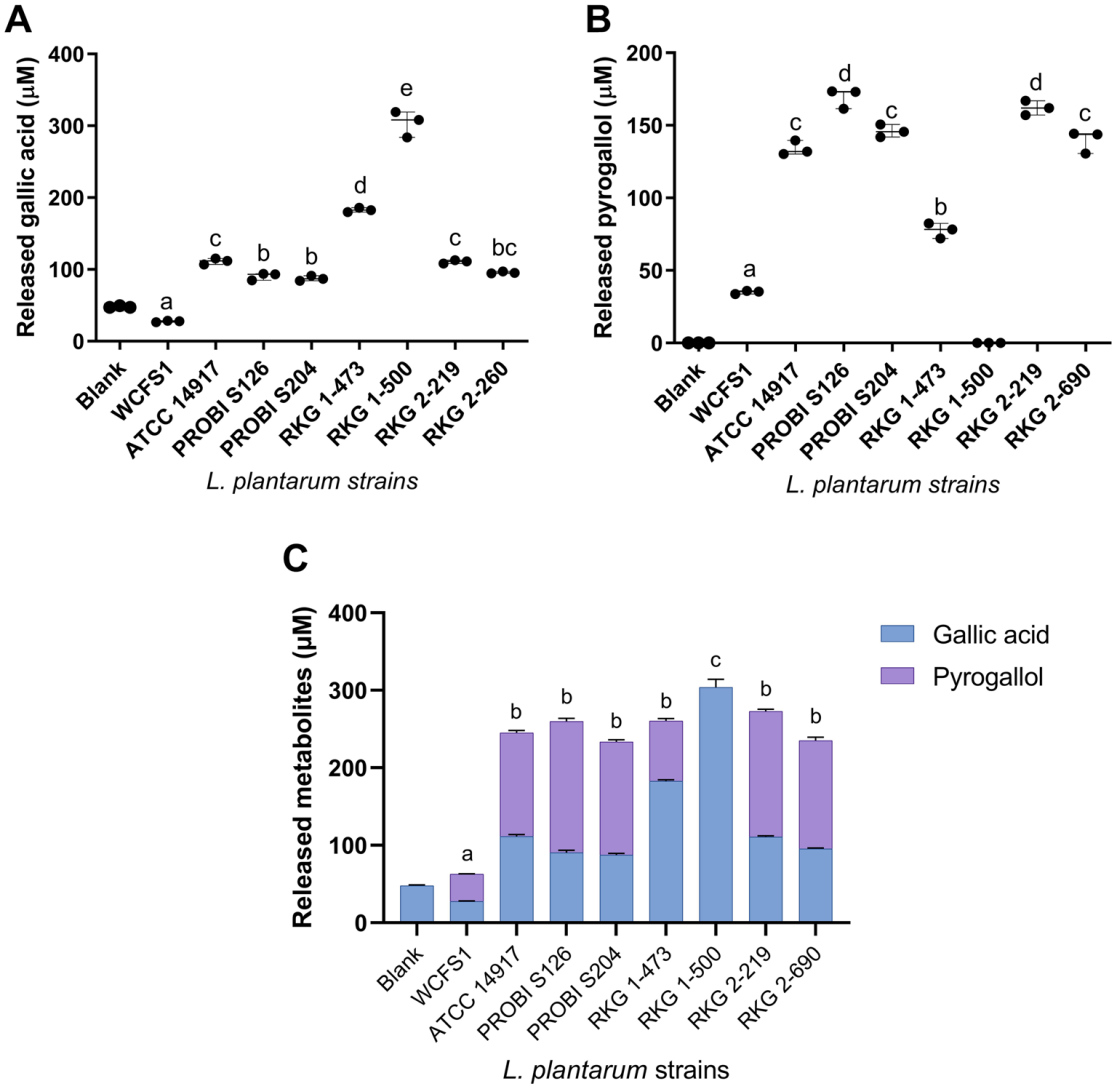
Phenolic metabolite-release by the selected TanA_+_ *L. plantarum* strains cultured in a tannic acid-rich medium. (**a**) Released gallic acid; (**b**) released pyrogallol; (**c**) total released metabolites. The values of three independent experiments or their mean ± SEM are shown. Different letters show statistical differences between strains at < *p* 0.01. Ordinary One-way ANOVA with Tukey’s post hoc test was used to compare strains. Strains that produced no detectable metabolites were removed from comparations. The blank (uninoculated media) contained a residual gallic acid from the tannic acid reagent that increased over time due to degradation at incubation conditions (30 °C).

The capacity of TanA+ strains to metabolize tannic acid was measured indirectly by quantifying the released metabolites (i.e., gallic acid and pyrogallol) by UPLC-UV-QToF. TanA+ strains released 3.2–11 times more gallic acid than the control TanA lacking strain (WCFS1) (p < 0.05) (Fig. 4a); those with gallate decarboxylase activity released 2.2–4.8 times more pyrogallol than the TanA lacking strain (p < 0.05) (Fig. 4b). Except for the strain RKG 1-473, all TanA+ strains exhibiting gallate decarboxylase activity showed a similar pattern of metabolite release: more than a third of total final metabolites stayed as gallic acid, while more than half were transformed into pyrogallol (Fig. 4c). *L. plantarum* RKG 1-500 was the strain that released more gallic acid (p < 0.0001) (Fig. 4a), as this strain lacks the gallate decarboxylate activity and therefore is unable to transform this metabolite further.

#### Transforming capacities of TanA+ strains towards punicalagin

The selected TanA+ strains were unable to transform the punicalagin supplemented to their culture medium. Indeed, punicalagin concentrations did not change significantly throughout the fermentation with a TanA+ (ATCC 14917) or a TanA lacking (WCFS1) reference strain (Fig. S4a, supplementary material). Moreover, on day 10, the punicalagin from the fermented culture media of different TanA+ strains did not differ either (*p* > 0.05) (Fig. S4b, supplementary material). Furthermore, the production of punicalagin-metabolites (i.e., ellagic acid and urolithins) was not observed.

### Growth rate of TanA+ strains in the presence of gallic acid and tannic acid (poly)phenol substrates

TanA+ strains were cultured in the presence of different concentrations of (poly)phenol substrates to identify growth patterns or possible differences between the growth responses of transforming and non-transforming strains. In most cases, adding tannic acid to the culture medium resulted in a reduced relative growth rate (% of µ_max_) of TanA+ strains (Table 1). Overall, the TanA lacking strain (WCFS1) was more susceptible to the tannic acid antimicrobial effect in the three tested concentrations (Table 1) (p < 0.05). Remarkably, the lowest tannic acid dose (5 µM) did not harm and even slightly promoted the relative growth rate of two TanA+ strains, PROBI S204 and RKG 2-219 (103.2 ± 2.7% and 111.2 ± 1.3%, respectively) (p < 0.05) (Table 1).

**Table 1:**
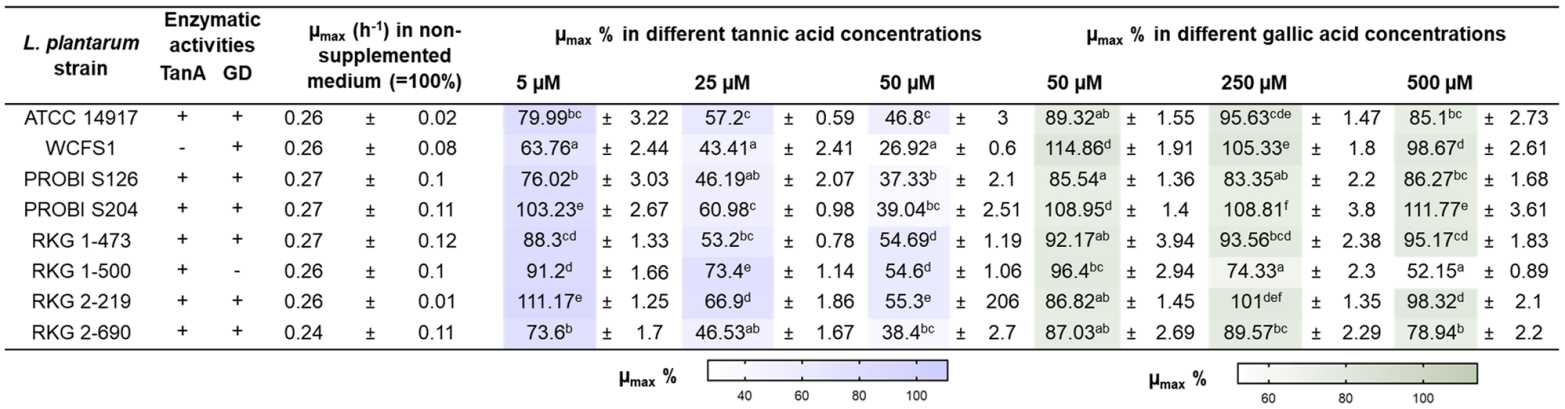
Growth response of *L. plantarum’*to the presence of tannic and gallic acid phenolics in a minimal growth medium.

Data in the same column with different letters are significantly different at < *p* 0.01. Statistical significance was determined by a two-way ANOVA (*p* < 0.05) followed by Tukey’s multiple comparisons test. Color intensity increases proportionally to the mean values. Heat map colors were obtained in GraphPad 9.4.0.

*ATCC* American type culture collection, *RKG* Roy, Kennang, and Gagnon collection, *TanA* extracellular tannase, *GD* gallate decarboxylase, *µ*_*max*_* %* relative growth rates.

Compared to tannic acid, gallic acid had a lower growth inhibitory effect (Table 1). This, except for the gallate decarboxylase lacking strain (RKG 1-500), which grew slower at the two highest gallic acid concentrations (250 and 500 µM) and presented a similar growth pattern than in the tannic acid supplemented media.

### Correlations of released (poly)phenol metabolites and growth rate of *L. plantarum* cultured with tannic acid

To explore possible relationships between the metabolism of TanA+ strains and their fitness, we performed correlations of the released metabolites and the growth rate of each strain (Fig. S5, supplementary material). A positive correlation (*p* = 0.0184, r = 0.7619) was found between the ability of the strains to release gallic acid and their growth rate when cultured in the tannic acid-supplemented medium (Fig. S5a, supplementary material). In contrast, pyrogallol release was not significantly correlated to the growth of *L. plantarum* (*p* = 0.9349, *r* = − 0.04762, Fig. S5b).

### Comparative genomic analysis of TanA+ strains

The six selected TanA+ strains genomes are available in the BV-BRC; their identification number and characteristics are described in Table S3 (supplementary material). These strains presented a genome size ranging between 3.14 and 3.29 Mbp. After assembly, these were marked as good quality genomes, as they show 100% completeness, over 97.7% of coarse consistency, and 95.8% of fine consistency (predicted with EvalG and EvalCon tools^36^).

**Figure 5:**
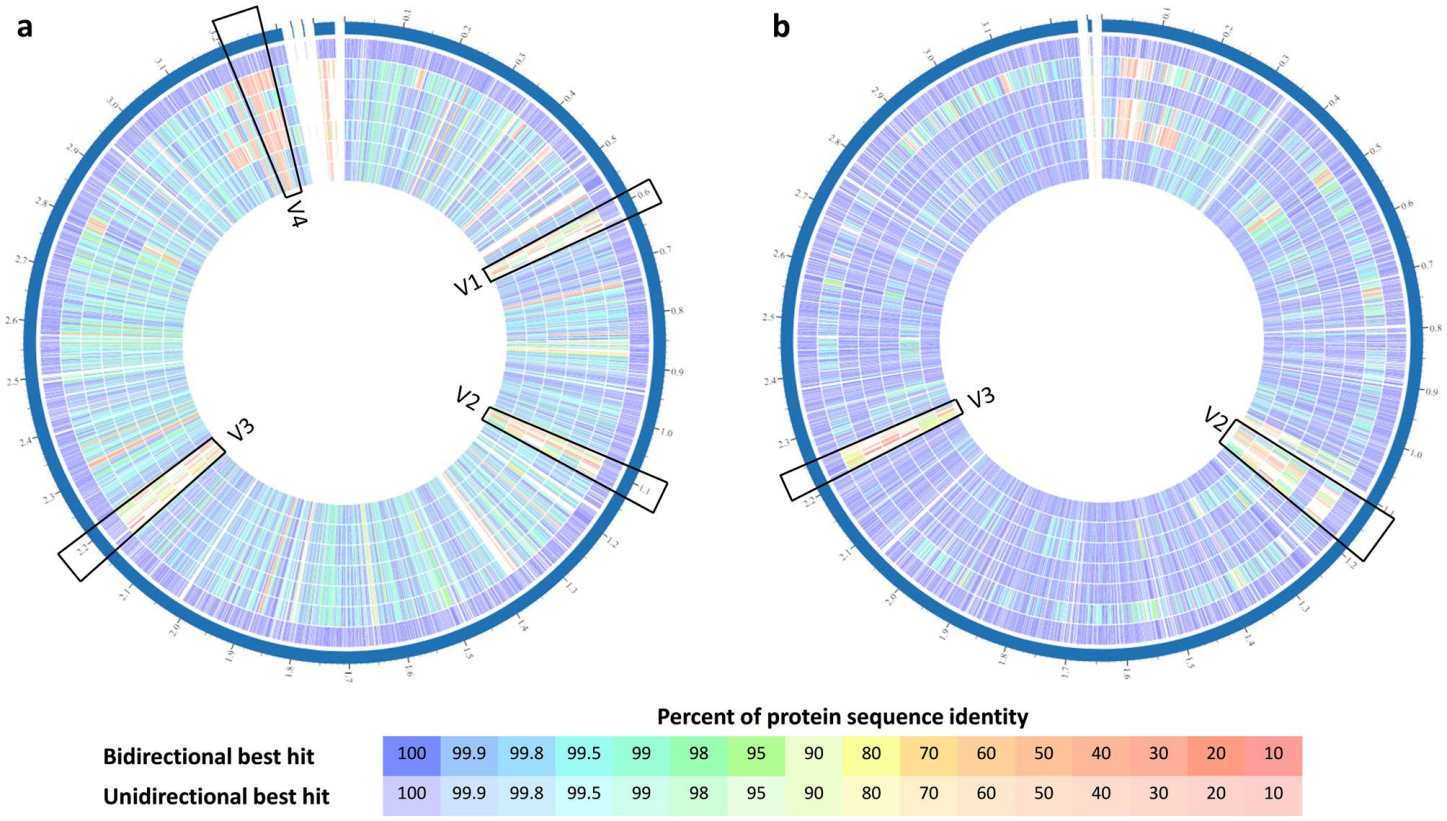
Comparison of the genomic features of the selected TanA+ strains with two reference *L. plantarum* strains. (**a**) With WCFS1 as reference strain, where four variable regions are distinguished (V1-V4); (**b**) with ATCC 14917 as reference strain, where two variable regions are perceived (V2 and V3). List of tracks, from outside to inside: **1**, reference strain; **2**, PROBI S126; **3**, PROBI S204; **4,** RKG 1-473; **5**, RKG 1-500; **6**, RKG 2-219; **7**, RKG 2-690.

The genomic features of TanA+ strains were compared with those of the TanA lacking reference strain (WCFS1), obtaining four regions of high variability as a result (Fig. 5a). These are two phage-associated regions: V1 (*lp_0624* to *lp_0631*) and V3 (*lp_2398* to *lp_2448*); a region related with surface polysaccharide synthesis: V2 (lp_1176 to lp_1233); and a region encoding proteins for sugar metabolism: V4 (*lp_3599* to *lp_3627*). In contrast, when ATCC 14917 (TanA+) strain was used as a reference strain, only V2 and V3 regions remained variable, indicating higher similarity between the proteome of TanA+ strains. In general, a greater identity of protein sequences between TanA+ strains and ATCC 14917 can be noticed (Fig. 5b). Moreover, we observed a remarkable resemblance between the PROBI S204 and the ATCC 14917 strain genomic features, for which variability is observed only within the V3 region.

### Genomic features encoding tannin-transforming enzymes

A multiple sequence alignment revealed that the six selected TanA+ strains have an identical TanA 626-aminoacid sequence (100% identity with ATCC 14917 strain). At the nucleotide level, four out of six strains showed identical sequences, whereas RKG 1-500 and RKG 2-219 showed a 708 C > T silent mutation (Fig. S6a, supplementary material). The gallate decarboxylase encoding genes, *lpdB* and *lpdC*, were present in all selected strains. However, in *L. plantarum* RKG 1-500, the *lpdC* gene is truncated, explaining why this strain lacks this enzymatic activity. The other five strains exhibiting gallate decarboxylase activity present identical LpdB and LpdC amino acid sequences, except for single substitutions: Glu-46 to Asp in RKG 2-219 LpdB sequence and Ala-115 to Ser in RKG 2-690 LpdC sequence (Fig. S6, supplementary material).

### Safety assessment of TanA+ selected strains based on their genome

The selected TanA+ strains share a profile of antimicrobial resistance genes (Table S4). The six strains harbor two sets of beta-lactamase genes (*penP*), a macrolide resistance gene (*msr, vmlR*), a tetracycline resistance gene (*tetM, tetO*), a phenicol resistance gene (*catA*), two vancomycin resistance genes (*vanX* and *VanY*) and several other genes related to efflux pumps, ATP-binding cassettes, and transcriptional regulators potentially conferring them multidrug resistances. Among all identified resistance genes, only one is located in a mobile element, an incomplete bacteriophage sequence (putatively defective) in the *L. plantarum* PROBI S126 genome (Table S5, supplementary material). The other antimicrobial resistance genes are situated in *L. plantarum* chromosomal DNA and were not flanked by transposal elements.

Except for the spermidine synthase gene [EC:2.5.1.16] (K00797 in the KEGG database), all the inspected biogenic amine production genes were absent in the studied genomes (Table S6, supplementary material). This feature was present in all the selected strains, excluding *L. plantarum* RKG 1-500.

### Phylogenetic analysis and characterization of tanA harboring strains according to their bacteriocin profile

**Figure 6:**
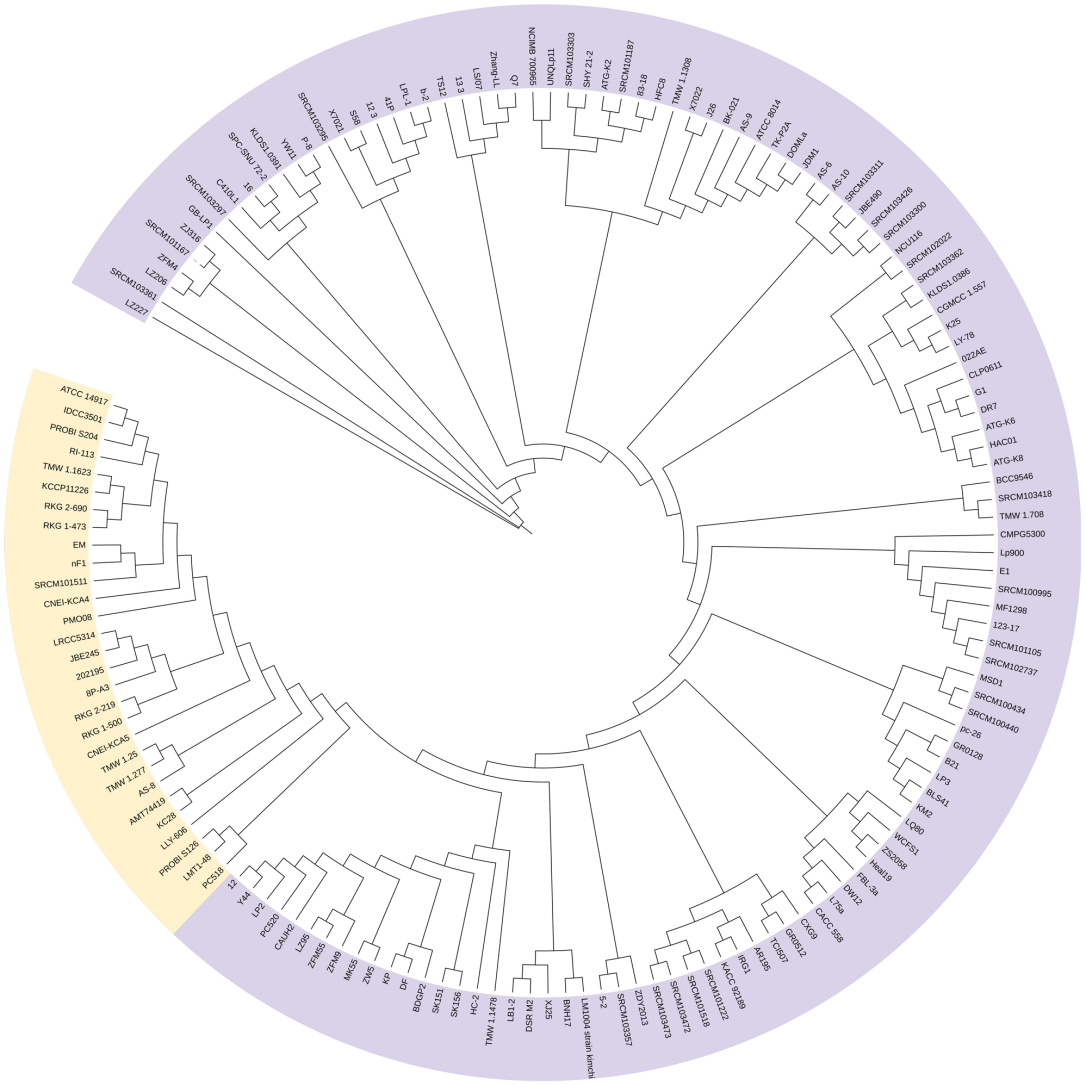
Phylogenetic tree of 155 *L. plantarum* strains. Genome sequences were obtained from BV-the BRC (149) and were analyzed with the ones obtained from the TanA+ strains selected in this study (6). In yellow, a cluster comprising 29 strains, two lacking the *tanA* gene (LLY-606 and PC518). The core genome-based phylogeny was performed by the randomized axelerated maximum likelihood in the BV-BRC. This image is available interactively at https://itol.embl.de/tree/142117188106405051646444270.

To understand the phylogenetic relationship between *L. plantarum tanA* harboring strains, a phylogenetic tree comprising 149 *L. plantarum* complete genomes and the ones of the six selected TanA+ strains was constructed (Fig. 6). Surprisingly, all *tanA* harboring strains form a specific cluster of 29 strains, in which only two lack this accessory gene (LLY-606 and PC518). Among *tanA* harboring strains, two bacteriocin gene patterns were distinguished (Fig. S7). The first pattern is characterized by the presence of PlnE, PlnF, PlnJ, and PlnN genomic features. In contrast, most strains presenting the second pattern have PlnE and PlnF but lack PlnJ and PlnN genomic features. The eventual presence of enterocin X chain β and NC8α and NC8β encoding genes was also observed in this group.

TanA activity was predicted in ten *tanA *harboring strains: LMT1-48, AS-8, CNEI-KCA5, JBE245, LRCC5314, ATCC 202195**,** 8P-A3, PMO08, CNEI-KCA4, IDCC3501; but is likely unfunctional in the others (Fig. S7a, supplementary material). All *tanA* harboring strains had the genes encoding the gallate decarboxylase (*lpdB*, *lpdC*). The multiple alignments of LpdB and LpdC amino acid sequences indicate that this enzymatic activity would be expected in most *tanA* harboring strains (16 out of 20), as their sequences match the ones observed by the gallate decarboxylase producing strains selected in this study (Fig. S7b,c, supplementary material). In contrast, TMW 1.25 and TMW 1.277 strains showed truncated lpdC amino acid sequences, and RI-113 and JBE245 LpdB specific mutations (Ala-154 to Ser and Pro-171 to Ser, respectively) that might interfere with their translation into functional gallate decarboxylase subunits.

### Other potential TanA+ species

A BLAST search using the ATCC 14917 TanA amino acid sequence as a query revealed other lactobacilli proteins producing significant alignments with this sequence. Indeed, sixteen tannase protein sequences from one closely related species, *Lactiplantibacillus pentosus*, shared more than 90% of identity with the query sequence. Likewise, proteins of species belonging to *Levilactobacillus,* such as *L. zymae, L. spicheri,* and *L. suantsaii* shared more than 80% of identity with the ATCC 14917 TanA amino acid sequence, indicating that other lactobacilli species might also produce extracellular tannases (Table S7, supplementary material).

## Discussion

A growing body of evidence underpins the use of transforming probiotic bacteria as valuable strategies to enhance the release of bioactive metabolites from (poly)phenols^37–40^. Pinpointing probiotic candidates with specific PAZymes that deliver the desired phenolic metabolites is of utmost importance to provide alternatives to subjects with unfavorable metabotypes. In this study, we present a practical approach to screen tannin-transforming *L. plantarum* strains and demonstrate their capacity to release gallotannin metabolites and grow in the presence of this phenolic substrate. The selected TanA+ *L. plantarum* strains are potentially safe and can be considered probiotic candidates for formulations aiming to potentiate (poly)phenols health effects.

The presence of an extracellular tannase in *L. plantarum* ATCC 14917 and its encoding gene (*tanA*, locus HMPREF0531_11477), was first reported by Jiménez et al.^5^. However, this gene was described as non-inducible, as its expression did not change after exposing *L. plantarum* cells to methyl-gallate (as substrate). Thus, it was not clear if the *tanA* steady expression would ultimately lead to a release of valuable amounts of gallotannins metabolites by this strain. In this study, we quantitatively characterized the time release of gallic acid and pyrogallol metabolites produced by *L. plantarum* ATCC 14917 from tannic acid and compared them with a TanA lacking strain (WCFS1). Released gallotannins metabolites became detectable at the beginning of the stationary growth phase, which indicates that the strain hydrolyzed the polymeric tannic acid molecules before this stage. The TanA+ reference strain attained the release of at least seven times more gallic acid than the TanA lacking reference strain, maintaining a nearly constant gallic acid concentration during the stationary growth phase, despite its simultaneous transformation to its ensuing metabolite, pyrogallol. This later phenolic metabolite was continuously released throughout the stationary growth phase, which suggests that gallic acid was used during this time as an alternative way of energy generation. Indeed, during gallic acid transformation, a proton motive force is produced by an energy generation system comprising the gallate decarboxylase, which intervenes in proton consumption, and an ion transporter (Lp_2943)^41^. All in all, *L. plantarum* ATCC 14917 showed a better release profile of both gallotannin metabolites than its counterpart TanA lacking reference strain (WCFS1).

According to our results, TanA activity is likely to be considered a desirable *L. plantarum* strain-specific probiotic feature, as it favors the release of highly bioactive phenolic metabolites. However, up to date, only three tannin-transforming *L. plantarum* strains (ATCC 14917, MTCC 1407, and CIR1) have been reported^5,42,43^. To enable TanA screening, we proposed a method targeting this extracellular activity in *L. plantarum*, which the available colorimetric techniques would not otherwise distinguish. Indeed, Nishitani and Osawa^44^ proposed an assay to quantify bacterial tannase activity; however, it uses methyl-gallate as a substrate, which both, intracellular and extracellular tannases, can metabolize. The method proposed herein is reliable and does not require sophisticated instrumentation, facilitating the screening of this enzymatic activity across *L. plantarum* culture collections. This method might also be suitable to screen TanA in other potentially producing lactobacilli such as *L. pentosus* and *Levilactobacillus* spp.

It has been proposed that PAZymes help bacteria detoxify themselves from the strong antimicrobial activity of phenolic compounds. But emerging evidence suggests that transformed (poly)phenols may also provide bacteria other advantages for their fitness^41,45–48^. In this study, we observed that TanA+ strains tended to grow faster in the presence of tannic acid when compared to a TanA lacking strain. Furthermore, the ability of the strains to release gallic acid was positively correlated with their growth rate. A possible explanation for this might be that during tannic acid metabolism, TanA+ strains gain access to the central glucose of this molecule and use it as a carbon source. However, it is noteworthy to underline that tannic acid resistance is highly strain-specific, as we observed differences among TanA+ strains that their phenolic transforming capacities would not explain. A plethora of strain-specific responses that allow bacteria to cope with (poly)phenols might be responsible for these variations; these are detoxification mechanisms (e.g., efflux pumps), genotoxic stress responses, and membrane and cell wall modifications, among others^49^.

Interestingly, we observed a similar response to tannic acid with gallic acid; gallate decarboxylase-producing strains had higher relative growth rates (µ_max_ %) than the gallate decarboxylase lacking strain. Gallic acid growth acceleration might result from an increased energy availability prompted by this phenolic acid by the mechanism mentioned above. In this manner, *L. plantarum,* with gallate decarboxylase activity, detoxifies its environment and gains an energetic advantage after being exposed to gallic acid. Other mechanisms might also be involved; for example, it has been demonstrated that gallic acid facilitates glucose and fructose uptake in *Lentilactobacillus hilgardii* 5w, which accelerates its growth when cultured in a gallic acid-rich medium^50^. Similar mechanisms might hold a place in *L. plantarum* metabolism in order to survive in (poly)phenol-rich harsh environments.

The dual effect of (poly)phenols on the growth of *L. plantarum* attracts particular attention: on the one hand, tannic acid triggered a stimulatory effect by providing a substrate that improved TanA+ *L. plantarum* fitness, and on the other, an antimicrobial effect that inhibited its growth. Albeit slighter, gallic acid exerted an antimicrobial effect on some strains while favoring the growth rate of those that produce the gallate decarboxylase enzyme. These facts illustrate the complexity when working with some (poly)phenol substrates as we address two opposite and indissociable effects. To acknowledge this phenomenon that is also widespread in the gut microbiota, Rodríguez-Daza et al. have recently proposed the term *duplibiotic*, which describes unabsorbed substrates modulating the gut microbiota by simultaneous antimicrobial and prebiotic modes of action^48^. Our results suggest that gallotannins (tannic acid) and gallic acid may fall within this category of compounds.

Notwithstanding TanA+ strains gallotannin hydrolyzing capacity, these were unable to break down the punicalagin molecule. However, this fact is not surprising as this transformation has only been reported in fungus, mainly in *Aspergillus* species^51^. Moreover, TanA_,_ and most bacterial tannases, share a low degree of similarity with fungal tannases^52^; hence differences in tannase substrate specificities are expected. The TanA of *L. plantarum* may be limited to acting on gallic acid and protocatechuic-derived esters^5^.

Nevertheless, it is essential to mention that culture conditions strongly impact bacteria metabolism. For instance, *Bifidobacterium pseudocatenulatum* INIA P815 can transform ellagic acid into urolithin A in BHI but not in RCM medium^53^. Likewise, compositional differences in the fermented medium, such as the amino acid and polysaccharide content, can compromise the accuracy when quantifying metabolites owing to interactions between these compounds and (poly)phenols^54^. Therefore, the minor differences observed between metabolite release by TanA+ strains could be explained by several factors other than differences in (poly)phenol metabolism, for example, differences between the amino acid metabolism and polysaccharide production of each strain, interactions between strain-specific capsular polysaccharides or surface proteins and (poly)phenols^54^, as well as the affinity of the strains to the culture medium. Yet, most of the TanA+ strains shared a metabolite release pattern, showing the reliability of our results as a whole, which portray the behavior of TanA+ *L. plantarum* strains.

Owing to the above-described reasons, it is difficult to highlight the performance of a specific strain. However, one evident metabolic difference between the selected TanA+ strains is the lack of gallate decarboxylase activity in one of them (RKG 1-500) due to a truncation in its *lpdC* gene. Furthermore, the analysis of the twenty available *tanA *harboring genomes showed that other strains, such as TMW 1.25 and TMW 1.277, also presented truncated *lpdC* genes, and therefore are likely to lack gallate decarboxylase activity. Because the presence of the *lpdB* and *lpdC* genes would not be useful for detecting this enzymatic activity, phenotypic tests should be conducted to assess the capacity of the strains. In our experience, the visual test proposed by Osawa and Wash (1995)^55^ is convenient and reliable for this purpose (data not shown). As mentioned, the gallate decarboxylase enzyme is desirable as it benefits the energetic gain and growth rate of *L. plantarum*. However, because this enzyme is highly widespread among gut bacteria and lactobacilli species^8,56^, it is unclear if its presence on *L. plantarum* impacts its in vivo health effect.

In this study, we show that TanA+ strains share a high identity percentage between their genomic features, showing a marked variability when compared to a TanA lacking strain. Indeed, the genomic features of TanA+ strains against those of the WCFS1 strain (lacking TanA) revealed four variable regions related to bacteriophages, polysaccharide production, and sugar metabolism. Strikingly, these same variable regions were found by Surve et al.^57^. when comparing the genomic features of the WCFS1 strain with those of two *L. plantarum* strains isolated from Indian foods (DKL3 and JGR2 from dhokla batter and jaggery) as well as those from other strains (ST-III, JDM1, P-8, 16, ZJ316, DSM 20174). In contrast, only two variable regions, encoding proteins for phage and sugar metabolism, are identified between TanA+ strains. These variable regions may be targeted to identify strain-specific features among the highly genetically related TanA+ *L. plantarum*.

The selected TanA+ strains also shared a profile of antimicrobial resistance genes. As expected, these strains harbour *vanX* and *VanY* genes, which confer vancomycin resistance, considered intrinsic in this species^58^. Other antimicrobial resistance genes were also detected, but according to our analysis, these have a low chance of being transferred and, therefore, do not pose a safety risk. Another point favouring the potential safety of the selected strains is that all genes encoding proteins involved in the biosynthesis of toxic biogenic amines are absent in their genomes. The only gene found in most strains codes for the spermidine synthase, which transforms putrescine into spermidine and spermine. Although speculative, this enzymatic activity might result in a positive outcome as it transforms a cytotoxin (i.e., putrescine)^59^ into beneficial polyamines (i.e., spermidine and spermine) with anti-aging^60^, anti-inflammatory, and pulmonary protecting activities^61^. Our genome analysis points to the selected TanA+ *L. plantarum* strains as potential safe ingredients or inoculants.

At the phylogenetic level, the tightly genetically related *tanA* harboring *L. plantarum* strains belong to the proposed lineage A, where they form a specific cluster. This finding suggests that PAZymes might also be good indicators of *L. plantarum* phylogeny. About half of the *tanA *harboring strains presented null mutations in this gene, possibly due to an adaptation through loss of function, during the evolution of these strains in tannin-lacking niches. These null mutations may revert if these strains re-encounter tannin-rich environments where this gene function seems beneficial^9^.

Finally, it is essential to mention that other tannase genes, homologous to *tanA* and *tanB* genes of *L. plantarum*, have been identified in pathogenic bacteria related to colorectal cancer (CRC), such as *Streptococcus gallolyticus*, *Staphylococcus lugdunensis* and *Fusobacterium nucleatum* subsp. *polymorphum*^62,63^. In CRC physiological conditions, the slow food flow leads to the accumulation of plant-derived compounds, such as tannins, creating a favourable niche for tannase-producing pathogenic bacteria^63^. By transforming tannins, such as tannic acid, the pathogenic bacteria abrogate the anti-proliferative effect exerted by this (poly)phenol on tumorous cells, boosting tumour progression^64^. Moreover, the product of this hydrolysis, gallic acid, might also play a role in CRC pathogenesis, as it has been observed it hyperactivates the WNT pathway in p-53 mutant organoids and mutant mice, conferring a malignant phenotype^65^. Although the information is still very scarce, the administration of tannase-producing probiotic bacteria might not be recommended in CRC.

## Conclusion

TanA is a strain-specific feature of *L. plantarum* that enable the transformation of gallotannins and contributes to *L. plantarum* fitness in tannin-rich niches. TanA allows *L. plantarum* to detoxify its environment from gallotannins, facilitating the uptake of gallic acid and tannin core carbon sources to support its survival and growth. Phylogenetically, TanA+ *L. plantarum* strains form a cluster inside the lineage A of the genetically heterogeneous *L. plantarum* species, sharing two profiles of bacteriocin coding sequences. As demonstrated in this study, TanA+ *L. plantarum* strains possess a specific ability to release bioactive metabolites from gallotannins. Considering the health potential of gallic and pyrogallol and other downstream metabolites such as catechol, the presence of TanA may be regarded as a desirable strain-specific *L. plantarum* probiotic feature. This functional feature can be detected by the screening approach proposed herein.

Future research is warranted to explore the in vivo potential health benefits of TanA+ *L. plantarum* strains. For instance, these strains may enhance colonic gallic acid and pyrogallol bioaccessibility by performing the first limiting transformation step of unabsorbed gallotannins (more than 60%^66,67^). By increasing the bioaccessibility of these phenolic metabolites, TanA+ strains may enhance the anti-obesity^68,69^, antidiabetic^69^, cardioprotective^70,71^, and neuroprotective effects^72,73^ of gallotannins.

## Supplementary Information

Supplementary Information.

## Data Availability

The genome sequences generated in this study can be accessed in the BV-BRC (https://www.bv-brc.org/) with the following ID: 1590.3023, 1590.3024, 1590.2643, 1590.2644, 1590.2648, 1590.2646. In addition, the produced and analyzed datasets will be available from the corresponding author upon reasonable request.
