# Supplementary material for: Comprehensive analysis of the metabolic and genomic features of tannin-transforming Lactiplantibacillus plantarum strains
Source: Sci Rep. 2022 Dec 27;12:22406. doi: 10.1038/s41598-022-26005-4 (PMC9794748; doi:10.1038/s41598-022-26005-4)
Supplement: Supplementary file 1 — Supplementary Information. [file 41598_2022_26005_MOESM1_ESM.docx]

Supplementary material

Comprehensive analysis of the metabolic and genomic features of tannin transforming *Lactiplantibacillus plantarum* strains

Elena C. Pulido-Mateos, Jacob Lessard-Lord, Denis Guyonnet, Yves Desjardins, and Denis Roy^*^

**Figure S1. Spectrophotometric method to screen TanA activity in *L. plantarum*. a**, representative visual reading obtained for blank, TanA lacking strain (WCFS1), and TanA+ strain (ATCC 14917). After tannic acid’s transformation, the released metabolites (gallic acid and pyrogallol) form a complex with the rhodanine molecule, producing a pink color. Visual color differences are perceived after 48 h of fermentation. **b**, Gallic acid calibration curve using the proposed spectrophotometric method to screen TanA activity in *L. plantarum.*

**Table S1. Genomes sequences used to construct the *L. plantarum* phylogenetic tree**

| **Genome ID in the BV-BRC** | **Genome Name** | **Isolation Source** | **Isolation country** |
| --- | --- | --- | --- |
|  |  |  |  |
| 1284663.3 | *L. plantarum* ZJ316 | Healthy newborn fecal sample; feces |  |
| 1300221.4 | *L. plantarum* DOMLa |  |  |
| 1304889.3 | *L. plantarum* CMPG5300 | Vagina of healthy woman | Belgium |
| 1327988.3 | *L. plantarum* 16 |  |  |
| 1590.1048 | *L. plantarum* strain SK151 | Kimchi | South Korea |
| 1590.1049 | *L. plantarum* strain DSR_M2 | Kimchi | South Korea |
| 1590.1155 | *L. plantarum* strain nF1 | Kimchi (traditional fermented korean dish) | South Korea |
| 1590.1159 | *L. plantarum* strain TMW 1.1478 | Honey | Germany |
| 1590.1167 | *L. plantarum* strain b-2 | Pickle | China |
| 1590.1168 | *L. plantarum* strain IDCC3501 | Kimchi | South Korea |
| 1590.1173 | *L. plantarum* strain DR7 | Milk | Malaysia |
| 1590.1174 | *L. plantarum* strain ZFM55 | Healthy infant fecal samples | China |
| 1590.1205 | *L. plantarum* strain ATG-K6 | Kimchi | South Korea |
| 1590.1206 | *L. plantarum* strain ATG-K8 | Kimchi | South Korea |
| 1590.1207 | *L. plantarum* strain ATG-K2 | Kimchi | South Korea |
| 1590.1208 | *L. plantarum* strain NCIMB 700965 | New Zealand cheese | New Zealand |
| 1590.1209 | *L. plantarum* strain ZFM9 | Healthy infant fecal samples | China |
| 1590.1210 | *L. plantarum* strain ZFM4 | Healthy infant fecal samples | China |
| 1590.1211 | *L. plantarum* strain KACC 92189 |  | South Korea |
| 1590.1214 | *L. plantarum* strain LMT1-48 | Kimchi | South Korea |
| 1590.1238 | *L. plantarum* strain FBL-3a | Feces | China |
| 1590.1239 | *L. plantarum* strain Q7 | Yak fermented milk | China |
| 1590.1377 | *L. plantarum* strain YW11 | Tibet kefir | China |
| 1590.1378 | *L. plantarum* strain 13_3 | Tibet kefir | China |
| 1590.1379 | *L. plantarum* strain 12_3 | Tibet kefir | China |
| 1590.1385 | *L. plantarum* strain SRCM103295 | Food | South Korea |
| 1590.1386 | *L. plantarum* strain SRCM103311 | Food | South Korea |
| 1590.1387 | *L. plantarum* strain SRCM103357 | Food | South Korea |
| 1590.1388 | *L. plantarum* strain SRCM103361 | Food | South Korea |
| 1590.1389 | *L. plantarum* strain SRCM103362 | Food | South Korea |
| 1590.1390 | *L. plantarum* strain SRCM103418 | Food | South Korea |
| 1590.1391 | *L. plantarum* strain SRCM103426 | Food | South Korea |
| 1590.1394 | *L. plantarum* strain SRCM103473 | Food | South Korea |
| 1590.1395 | *L. plantarum* strain SRCM103472 | Food | South Korea |
| 1590.1400 | *L. plantarum* strain SRCM103297 | Food | South Korea |
| 1590.1401 | *L. plantarum* strain SRCM103300 | Food | South Korea |
| 1590.1402 | *L. plantarum* strain SRCM103303 | Food | South Korea |
| 1590.144 | *L. plantarum* strain B21 | Vietnamese fermented sausage (nem chua) | Viet Nam |
| 1590.1495 | *L. plantarum* strain IRG1 | Korean infant feces | South Korea |
| 1590.1497 | *L. plantarum* strain EM | Kimchi | South Korea |
| 1590.1502 | *L. plantarum* strain UNQLp11 | Pinot noir wine | Argentina |
| 1590.1503 | *L. plantarum* strain J26 | Traditional fermented dairy products in Inner Mongolia of China | China |
| 1590.152 | *L. plantarum* strain 5-2 | Fermented soybean | China |
| 1590.153 | *L. plantarum* strain ZS2058 | Sauerkraut | China |
| 1590.154 | *L. plantarum* strain HFC8 |  | India |
| 1590.1541 | *L. plantarum* strain pc-26 | Healthy adult fecal sample | China |
| 1590.1542 | *L. plantarum* strain LLY-606 | Healthy adult fecal sample | China |
| 1590.1543 | *L. plantarum* strain Y44 | Turbot | China |
| 1590.1589 | *L. plantarum* strain TMW 1.1308 |  | Germany |
| 1590.1603 | *L. plantarum* strain KCCP11226 | Kimchi (Korean traditional fermented food) | South Korea |
| 1590.1605 | *L. plantarum* strain 83-18 | Human feces (woman, 60 years old) | Russia |
| 1590.1606 | *L. plantarum* strain 123-17 | Human feces (woman, 24 years old) | Russia |
| 1590.1630 | *L. plantarum* strain 8P-A3 | Probiotic preparation "Lactobacterinum" | Russia |
| 1590.1656 | *L. plantarum* strain SRCM100440 | Infant faecal | South Korea |
| 1590.1658 | *L. plantarum* strain SRCM100995 | Pickled green chili peppers | South Korea |
| 1590.1659 | *L. plantarum* strain SRCM101105 | Kimchi | South Korea |
| 1590.1660 | *L. plantarum* strain SRCM101187 | Kimchi without red pepper powder | South Korea |
| 1590.1661 | *L. plantarum* strain SRCM101222 | Young radish kimchi | South Korea |
| 1590.1662 | *L. plantarum* strain SRCM101518 | Young radish kimchi | South Korea |
| 1590.1663 | *L. plantarum* strain SRCM102737 | Soybean paste (Chonggugjang) | South Korea |
| 1590.1664 | *L. plantarum* strain SRCM101167 | Water kimchi | South Korea |
| 1590.1695 | *L. plantarum* strain SRCM101511 | Diced radish kimchi | South Korea |
| 1590.1696 | *L. plantarum* strain CACC 558 | Canine feces | South Korea |
| 1590.1699 | *L. plantarum* strain X7022 |  | China |
| 1590.1702 | *L. plantarum* strain LS/07 |  | Slovakia |
| 1590.1708 | *L. plantarum* strain SPC-SNU 72-2 | Kimchi | South Korea |
| 1590.1744 | *L. plantarum* strain AMT74419 | Kimchi | South Korea |
| 1590.1745 | *L. plantarum* strain CNEI-KCA4 | Fermented okpei-onitsha | Nigeria |
| 1590.175 | *L. plantarum* strain LZ95 | Baby stool | China |
| 1590.1767 | *L. plantarum* strain TCI507 | Orange | Taiwan |
| 1590.1769 | *L. plantarum* strain Heal19 | Human GI tract | Sweden |
| 1590.1782 | *L. plantarum* strain HC-2 |  | China |
| 1590.1783 | *L. plantarum* strain BK-021 | Fermented onions | South Korea |
| 1590.1796 | *L. plantarum* strain CNEI-KCA5 | Fermented okpei-nsukka | Nigeria |
| 1590.1801 | *L. plantarum* strain SK156 | Fermented food | South Korea |
| 1590.1802 | *L. plantarum* strain BCC9546 | Fermented pork (nham) | Thailand |
| 1590.1803 | *L. plantarum* strain DSM 20174 (or ATCC 14917) | Pickled cabbage |  |
| 1590.185 | *L. plantarum* strain Zhang-LL | Fermented rice |  |
| 1590.192 | *L. plantarum* strain JBE245 | Meju, fermented soybean paste | South Korea |
| 1590.195 | *L. plantarum* strain CAUH2 | Sichuan pickle vegetables | China |
| 1590.2020 | *L. plantarum* strain PMO08 | Kimchi | South Korea |
| 1590.2024 | *L. plantarum* strain TK-P2A | Probitic products | China |
| 1590.2069 | *L. plantarum* strain MK55 |  | Turkey |
| 1590.2070 | *L. plantarum* strain ZDY2013 | Traditional Chinese fermented soybeans | China |
| 1590.2074 | *L. plantarum* strain SHY 21-2 | Sichuan red original yak yogurt | China |
| 1590.2089 | *L. plantarum* strain PC518 |  | China |
| 1590.2125 | *L. plantarum* strain S58 | Chinese pickle | China |
| 1590.2126 | *L. plantarum* strain CXG9 | Stinky xiancaigeng | China |
| 1590.2127 | *L. plantarum* strain KM2 | Ripening beef | South Korea |
| 1590.2128 | *L. plantarum* strain Lp900 | Ogi (red sorghum) | Nigeria |
| 1590.2145 | *L. plantarum* strain GR0512 | Fu-Tsai (Fermented vegetable, food) | Taiwan |
| 1590.2146 | *L. plantarum* strain GR0128 | Fu-Tsai (Fermented vegetable, food) | Taiwan |
| 1590.2147 | *L. plantarum* strain 12 |  | China |
| 1590.2148 | *L. plantarum* strain AR195 | Rice wine rice syrup | China |
| 1590.2149 | *L. plantarum* strain KLDS1.0386 | Traditional fermented dairy products in Inner Mongolia | China |
| 1590.2186 | *L. plantarum* strain LRCC5314 | Kimchi | South Korea |
| 1590.2253 | *L. plantarum* strain ATCC 202195 | Healthy infant feces | USA |
| 1590.2305 | *L. plantarum* strain XJ25 | Wine | China |
| 1590.2309 | *L. plantarum* strain L75a | South china sea | China |
| 1590.2316 | *L. plantarum* strain 41P | Meat | Ireland |
| 1590.2317 | *L. plantarum* strain DW12 | Fermented food | Thailand |
| 1590.2318 | *L. plantarum* strain MSD1 | Curd | India |
| 1590.2553 | *L. plantarum* ZW5 | Water | China |
| 1590.2568 | *L. plantarum* 022AE | Fermented dairy sample | India |
| 1590.265 | *L. plantarum* strain LZ206 | Raw cow milk | China |
| 1590.266 | *L. plantarum* strain LZ227 | Raw cow milk | China |
| 1590.269 | *L. plantarum* strain NCU116 | Nanchang | China |
| 1590.287 | *L. plantarum* strain KP | Whole fly | Canada |
| 1590.288 | *L. plantarum* strain DF | Whole fly | Canada |
| 1590.293 | *L. plantarum* strain LY-78 | Fermented chinese cabbage | China |
| 1590.310 | *L. plantarum* strain C410L1 | Pit mud | China |
| 1590.323 | *L. plantarum* strain MF1298 | Fermented sausage, Norway | Norway |
| 1590.384 | *L. plantarum* strain RI-113 | Fermented salami |  |
| 1590.386 | *L. plantarum* strain CLP0611 | Environment |  |
| 1590.465 | *L. plantarum* strain KLDS1.0391 |  | China |
| 1590.466 | *L. plantarum* strain JBE490 | Nuruk, Korean traditional beverage starter | South Korea |
| 1590.467 | *L. plantarum* strain LP2 | Chinese traditional pickles | China |
| 1590.468 | *L. plantarum* strain BLS41 | Kimchi | South Korea |
| 1590.469 | *L. plantarum* strain TMW 1.25 | Raw sausage | Germany |
| 1590.470 | *L. plantarum* strain TMW 1.277 | Palm wine | Germany |
| 1590.471 | *L. plantarum* strain TMW 1.708 | Raw sausage | Germany |
| 1590.472 | *L. plantarum* strain TMW 1.1623 |  | Germany |
| 1590.481 | *L. plantarum* strain SRCM102022 | Food | South Korea |
| 1590.482 | *L. plantarum* strain LPL-1 | Fermented fish | China |
| 1590.548 | *L. plantarum* strain LP3 | Commercial dietary supplements | South Korea |
| 1590.549 | *L. plantarum* strain BDGP2 | The feces of adult Drosophila melanogaster, Oregon-R-modencode strain | USA |
| 1590.551 | *L. plantarum* strain GB-LP1 |  | South Korea |
| 1590.601 | *L. plantarum* strain PC520 | Chinese fermented food-pickles | China |
| 1590.619 | *L. plantarum* strain ATCC 8014 | Unkown |  |
| 1590.914 | *L. plantarum* strain LM1004 strain kimchi | Napa cabbage kimchi | South Korea |
| 1590.917 | *L. plantarum* strain X7021 | Brine of stinky tofu | China |
| 1590.918 | *L. plantarum* strain KC28 | Fermented kimchi | South Korea |
| 1590.930 | *L. plantarum* strain HAC01 | White kimchi (baek kimchi) | South Korea |
| 1590.931 | *L. plantarum* strain LQ80 | Pig feed from feed plant | Japan |
| 1590.939 | *L. plantarum* strain AS-6 | Fruits and Vegetables | Pakistan |
| 1590.940 | *L. plantarum* strain AS-9 | Fruits and Vegetables | Pakistan |
| 1590.941 | *L. plantarum* strain AS-8 | Fruits and Vegetables | Pakistan |
| 1590.942 | *L. plantarum* strain AS-10 | Fruits and Vegetables | Pakistan |
| 1590.944 | *L. plantarum* strain K25 | Fermented milk | China |
| 220668.9 | *L. plantarum* WCFS1 | Human saliva |  |
| 337330.18 | *L. plantarum* subsp. *plantarum* strain TS12 | Stinky tofu | Malaysia |
| 337330.19 | *L. plantarum* subsp. *plantarum* strain SRCM100434 | Food | South Korea |
| 337330.36 | *L. plantarum* subsp. *plantarum* strain LB1-2 | Apis mellifera hindgut | Philippines |
| 337330.40 | *L. plantarum* subsp. *plantarum* strain E1 | Eup (fermented bamboo shoot) | India |
| 337330.68 | *L. plantarum* subsp. *plantarum* strain BNH17 | Angelica gigas Nakai root surface | South Korea |
| 337330.72 | *L. plantarum* subsp. *plantarum* strain G1 | Wheat germ | Brazil |
| 337330.9 | *L. plantarum* subsp. *plantarum* strain CGMCC 1.557 |  | China |
| 644042.3 | *L. plantarum* JDM1 |  |  |
| 767468.48 | *L. plantarum* subsp. *plantarum* P-8 | Plant material, and the gastrointestinal tract of animals |  |

**Figure S2. *L. plantarum* ATCC 14917 growth on RP-M basal medium supplemented with tannic acid (50 µM)**

**Table S2.** ***L. plantarum* isolates screened for *tanA* gene**

| Culture Collection | Isolate | Isolation source | Country of origin | *tanA* gene |  | TanA activity |
| --- | --- | --- | --- | --- | --- | --- |
| Probi | 6A | Human rectum | Sweden | - |  | - |
|  | 12C | Human, oral | Sweden | - |  | - |
|  | 12D | Human, oral | Sweden | - |  | - |
|  | 32A | Human, oral | Sweden | - |  | - |
|  | 35C | Human, oral | Sweden | - |  | - |
|  | 36D | Human, oral | Sweden | - |  | - |
|  | 37C | Human, oral | Sweden | - |  | - |
|  | 39-26 | Fermented sorghum | Sweden | - |  | - |
|  | 39-27 | Sorghum | Sweden | - |  | - |
|  | 42A | Human rectum | Sweden | - |  | - |
|  | 49-26 | Fermented sorghum | Sweden | - |  | - |
|  | 49-27 | Fermented sorghum | Sweden | - |  | - |
|  | 56-12 | Fermented sorghum | Sweden | - |  | - |
|  | 56-24 | Fermented sorghum | Sweden | - |  | - |
|  | 59-12 | Fermented sorghum | Sweden | - |  | - |
|  | 62C | Human, oral | Sweden | - |  | - |
|  | 67B | Human rectum | Sweden | - |  | - |
|  | 74-27 | Fermented sorghum | Sweden | - |  | - |
|  | 74-29 | Fermented sorghum | Sweden | - |  | - |
|  | 78B | Human, oral | Sweden | - |  | - |
|  | 81D | Human, oral | Sweden | - |  | - |
|  | 86C | Human, oral | Sweden | - |  | - |
|  | 88C | Human, oral | Sweden | - |  | - |
|  | 90-29 | Fermented sorghum | Sweden | - |  | - |
|  | 90-26 | Fermented sorghum | Sweden | - |  | - |
|  | 962 | Unknown | Sweden | - |  | - |
|  | E12 | Human feces | United Kingdom | - |  | - |
|  | I123 | Human feces | Italy | - |  | - |
|  | I154 | Human feces | Italy | - |  | - |
|  | I177 | Human feces | Italy | - |  | - |
|  | S71 | Human feces | Sweden | - |  | - |
|  | S114 | Human feces | Sweden | - |  | - |
|  | S126 | Human feces | Sweden | + |  | + |
|  | S144 | Human feces | Sweden | - |  | - |
|  | S177 | Human feces | Sweden | - |  | - |
|  | S204 | Human feces | Sweden | + |  | + |
|  | S265 | Human feces | Sweden | - |  | - |
|  | S268 | Human feces | Sweden | - |  | - |
| Roy, Kennang, and Gagnon | RKG 1-47 | Bovine raw milk | Canada | - |  | - |
|  | RKG 1-66 | Corn silage | Canada | - |  | - |
|  | RKG 1-116 | Grass silage | Canada | - |  | - |
|  | RKG 1-120 | Grass silage | Canada | - |  | - |
|  | RKG 1-122 | Grass silage | Canada | - |  | - |
|  | RKG 1-123 | Grass silage | Canada | - |  | - |
|  | RKG 1-124 | Grass silage | Canada | - |  | - |
|  | RKG 1-134 | Bovine raw milk | Canada | - |  | - |
|  | RKG 1-139 | Bovine raw milk | Canada | - |  | - |
|  | RKG 1-140 | Bovine raw milk | Canada | - |  | - |
|  | RKG 1-174B | Hay | Canada | - |  | - |
|  | RKG 1-176 | Hay | Canada | - |  | - |
|  | RKG 1-178 | Corn silage | Canada | - |  | - |
|  | RKG 1-270 | Corn silage | Canada | - |  | - |
|  | RKG 1-272 | Corn silage | Canada | - |  | - |
|  | RKG 1-292 | Grass silage | Canada | - |  | - |
|  | RKG 1-294 | Grass silage | Canada | - |  | - |
|  | RKG 1-329 | Corn silage | Canada | - |  | - |
|  | RKG 1-336 | Corn silage | Canada | - |  | - |
|  | RKG 1-339 | Corn silage | Canada | - |  | - |
|  | RKG 1-349 | Grass silage | Canada | - |  | - |
|  | RKG 1-351 | Corn silage | Canada | - |  | - |
|  | RKG 1-353 | Corn silage | Canada | - |  | - |
|  | RKG 1-354 | Corn silage | Canada | - |  | - |
|  | RKG 1-355 | Corn silage | Canada | - |  | - |
|  | RKG 1-358 | Corn silage | Canada | - |  | - |
|  | RKG 1-367 | Grass silage | Canada | - |  | - |
|  | RKG 1-371 | Bovine raw milk | Canada | - |  | - |
|  | RKG 1-375 | Bovine raw milk | Canada | - |  | - |
|  | RKG 1-383 | Corn silage | Canada | - |  | - |
|  | RKG 1-460 | Corn silage | Canada | - |  | - |
|  | RKG 1-464 | Corn silage | Canada | - |  | - |
|  | RKG 1-472 | Corn silage | Canada | - |  | - |
|  | RKG 1-473 | Corn silage | Canada | + |  | + |
|  | RKG 1-478 | Bovine raw milk | Canada | - |  | - |
|  | RKG 1-500 | Bovine raw milk | Canada | + |  | + |
|  | RKG 1-506 | Bovine raw milk | Canada | - |  | - |
|  | RKG 1-584 | Bovine raw milk | Canada | - |  | - |
|  | RKG 1-585 | Bovine raw milk | Canada | - |  | - |
|  | RKG 1-594 | Bovine raw milk | Canada | - |  | - |
|  | RKG 1-611 | Corn silage | Canada | + |  | - |
|  | RKG 1-612 | Corn silage | Canada | - |  | - |
|  | RKG 1-634 | Corn silage | Canada | - |  | - |
|  | RKG 2-13 | Corn silage | Canada | - |  | - |
|  | RKG 2-33 | Grass silage | Canada | - |  | - |
|  | RKG 2-75 | Bovine raw milk | Canada | - |  | - |
|  | RKG 2-211 | Bovine raw milk | Canada | - |  | - |
|  | RKG 2-212 | Bovine raw milk | Canada | - |  | - |
|  | RKG 2-219 | Corn silage | Canada | + |  | + |
|  | RKG 2-222 | Corn silage | Canada | - |  | - |
|  | RKG 2-227 | Corn silage | Canada | - |  | - |
|  | RKG 2-229 | Corn silage | Canada | - |  | - |
|  | RKG 2-273 | Grass silage | Canada | - |  | - |
|  | RKG 2-309 | Grass silage | Canada | - |  | - |
|  | RKG 2-361 | Bovine raw milk | Canada | - |  | - |
|  | RKG 2-384 | Grass silage | Canada | - |  | - |
|  | RKG 2-439 | Bovine raw milk | Canada | + |  | - |
|  | RKG 2-444 | Corn silage | Canada | - |  | - |
|  | RKG 2-448 | Corn silage | Canada | - |  | - |
|  | RKG 2-449 | Corn silage | Canada | - |  | - |
|  | RKG 2-512 | Corn silage | Canada | - |  | - |
|  | RKG 2-571 | Corn silage | Canada | - |  | - |
|  | RKG 2-523 | Grass silage | Canada | - |  | - |
|  | RKG 2-644 | Hay | Canada | - |  | - |
|  | RKG 2-648 | Hay | Canada | - |  | - |
|  | RKG 2-650 | Hay | Canada | - |  | - |
|  | RKG 2-664 | Hay | Canada | - |  | - |
|  | RKG 2-671 | Grass | Canada | - |  | - |
|  | RKG 2-676 | Corn silage | Canada | - |  | - |
|  | RKG 2-683 | Herb | Canada | - |  | - |
|  | RKG 2-684 | Herb | Canada | - |  | - |
|  | RKG 2-685 | Herb | Canada | - |  | - |
|  | RKG 2-687 | Grass silage | Canada | - |  | - |
|  | RKG 2-688 | Grass silage | Canada | - |  | - |
|  | RKG 2-690 | Grass silage | Canada | + |  | + |
|  | RKG 2-691 | Grass silage | Canada | - |  | - |
|  | RKG 2-693 | Grass silage | Canada | - |  | - |

TanA, extracellular tannase. GD, gallate decarboxylase.

**Figure S3. Pyrogallol concentrations at the end of the fermentation of a gallic acid-enriched medium.** The values of three independent experiments are shown.

**Figure S4. Punicalagin concentration in a minimal medium incubated with a TanA lacking and Tan+ *L. plantarum* strains. a,** Evolution of punicalagin throughout the fermentation with ATCC 14917 strain (TanA+, ▲); and WCFS1 strain (lacking TanA, ⚫,); as well as uninoculated punicalagin supplemented media (◼). A two-way repeated-measures ANOVA with the Geisser–Greenhouse correction followed by Dunnett post hoc test was used to compare groups. **b**, Punicalagin concentration at the end of the fermentation with different *L. plantarum* strains. Statistical significance was determined by using one-way ANOVA (*p*<0.05), followed by Holm-Šídák's multiple comparisons test to compare each TanA+ strain with the TanA lacking strain (WCFS1).

**Figure S5. Correlation of released phenolic metabolites with the growth rate of *L. plantarum* growth rate in tannic acid-rich medium (50 µM).** **a**, Correlation between released gallic acid and *L. plantarum*’s maximum growth rate**, b,** Correlation between released pyrogallol and *L. plantarum*’s growth rate**.** Spearman’s correlation coefficient r is indicated; a positive value indicates a positive correlation. In ▲, tanA lacking strain (WCFS1); in ⚫, TanA+ strains with gallate decarboxylase activity; in ◼, RKG 1‑500, TanA+ strain lacking gallate decarboxylase activity.

**Table S3. Characteristics of the whole-genome sequences of the selected *L. plantarum* TanA+ strains**

| ***L. plantarum* strain** | **PROBI S126** | **PROBI S204** | **RKG 1-473** | **RKG 1-500** | **RKG 2-219** | **RKG 2-690** |
| --- | --- | --- | --- | --- | --- | --- |
| **Genome ID in the BV-BRC** | 1590.3023 | 1590.3024 | 1590.2643 | 1590.2644 | 1590.2648 | 1590.2646 |
| **Size (bp)** | 3253101 | 3291059 | 3156267 | 3135602 | 3170607 | 3229984 |
| **Contigs** | 28 | 6 | 1 | 34 | 3 | 1 |
| **Coarse consistency predicted by EvalCon** | 97.8 | 97.7 | 97.9 | 97.8 | 97.9 | 97.8 |
| **Fine consistency predicted by EvalCon** | 96.5 | 95.8 | 96.2 | 95.8 | 96.2 | 95.9 |
| **Completeness (EvalG, %)** | 100 | 100 | 100 | 100 | 100 | 100 |
| **Contamination (CheckM, %)** | 0 | 0 | 0 | 0.2 | 0 | 0 |
| **Contigs N50 (bp)** | 3110648 | 3266832 | 3156267 | 2994037 | 3167262 | 3229984 |
| **Contigs L50** | 1 | 1 | 1 | 1 | 1 | 1 |
| **ANIm*, [aligned nucleotides], (%)** | 99.19 [86.99] | 99.23 [90.03] | 99.15 [92.43] | 99.16 [88.70] | 99.24 [91.96] | 99.31 [91.04] |
| **Coding DNA sequences in the BV-BRC** | 3239 | 3227 | 3088 | 3116 | 3086 | 3166 |
| **Putative plasmidic contigs ID in the BV-BRC** | 1590.2892 | 1590.2877 | 1590.2900 | 1590.2899 | Not found | 1590.2901 |

* Using *L. plantarum* WCFS1 as reference strain. The sequences were aligned using MUMmer.

**a**

*tanA* multiple sequence alignment


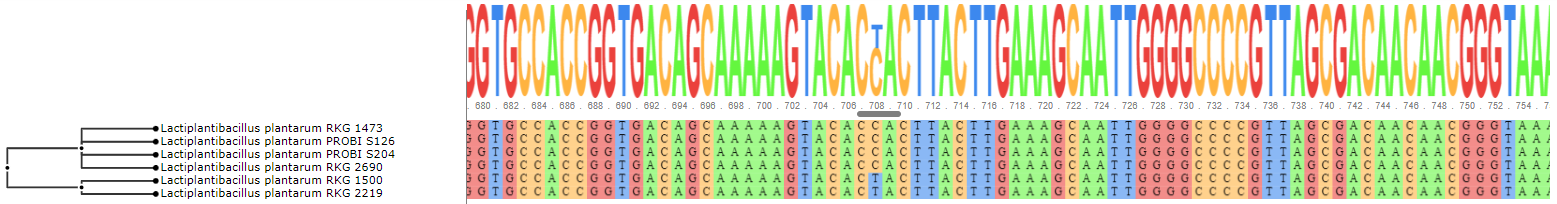


**b**

TanA multiple sequence alignment

*
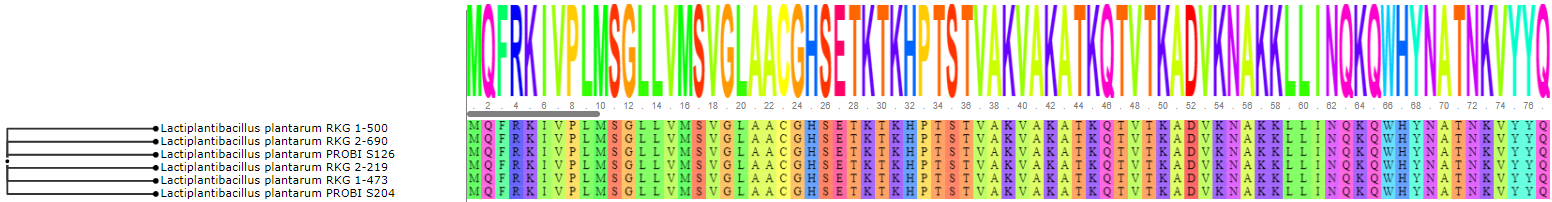
*

**c**

*lpdB* multiple sequence alignment


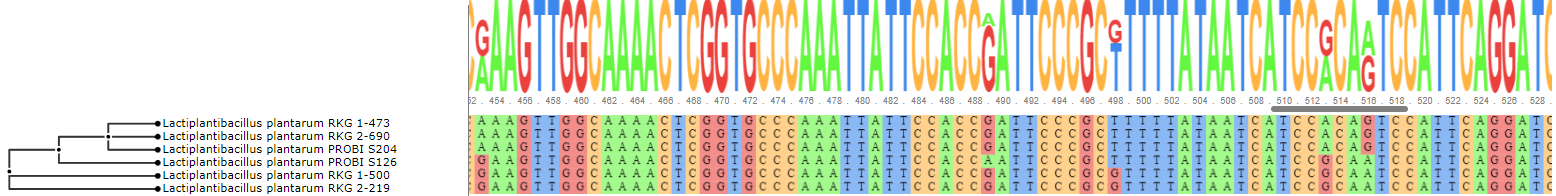


**d**

LpdB multiple sequence alignment


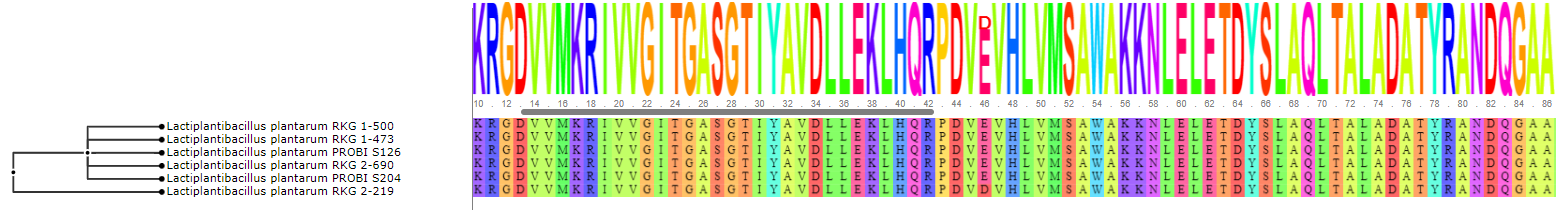


**e**

*lpdC* multiple sequence alignment
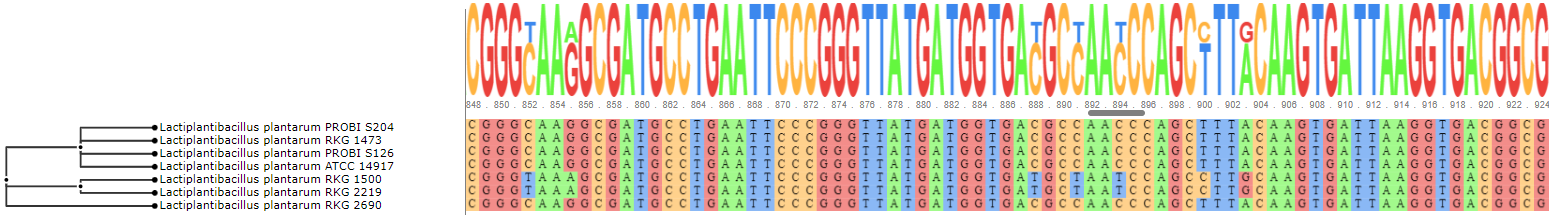


**f**

LpdC multiple sequence alignment
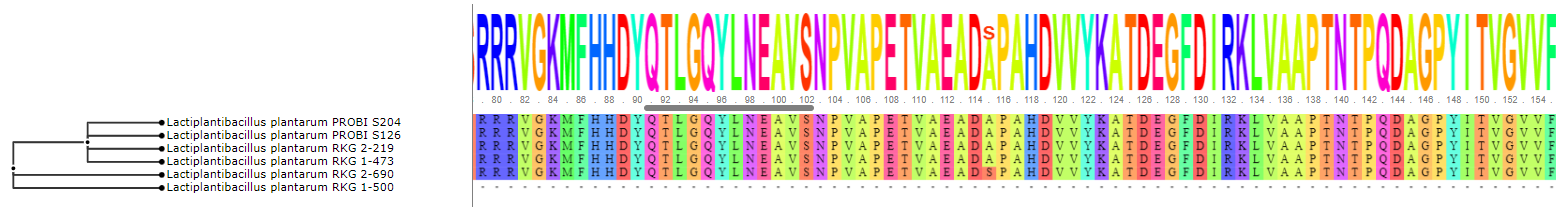


**Figure S6. Multiple sequence alignment of the genomic features encoding tannin-transforming enzymes in the selected TanA+ *L. plantarum* strains, performed in the BV-BRC**

**Table S4. Antibiotic resistance features in the *L. plantarum* selected strains**

| **Antibiotic resistance features** | | | ***L. plantarum* strain** | | | | | |
| --- | --- | --- | --- | --- | --- | --- | --- | --- |
| **Type of antibiotic resistance** | **KEGG_ID**  ^1,2,3D^ | **Gene Name and function** | **PROBI S126 feature (location)** | **PROBI S204**  **feature (location)** | **RKG 1-473**  **feature (location)** | **RKG 1-500**  **feature (location)** | **RKG 2-219**  **feature (location)** | **RKG 2-690**  **feature (location)** |
| **Aminoglycoside resistance** | [K05593](https://www.kegg.jp/entry/K05593) | aadK; aminoglycoside 6-adenylyltransferase [EC:2.7.7.-] |  |  |  | fig\|1590.2644.peg.2998  5097..5945 |  |  |
| **beta-Lactam resistance** | [K17836](https://www.kegg.jp/entry/K17836) | penP; beta-lactamase class A [EC:3.5.2.6] | fig\|1590.2604.peg.2003  (2049596..2050726) | fig\|1590.2594.peg.1056  (1068727..1069857) | fig\|1590.2643.peg.2417  (2455316..2456140) | fig\|1590.2644.peg.2513  (2552436..2553260) | fig\|1590.2648.peg.968  (1001971..1003101) | fig\|1590.2646.peg.1032  (1050789..1051919) |
|  | [K17836](https://www.kegg.jp/entry/K17836) | penP; beta-lactamase class A [EC:3.5.2.6] | fig\|1590.2604.peg.451  (459334..460158) | fig\|1590.2594.peg.2733  (2764480..2765304) | fig\|1590.2643.peg.740  (757591..758721) | fig\|1590.2644.peg.1004  (997942..999072) | fig\|1590.2648.peg.2629  (2685895..2686719) | fig\|1590.2646.peg.2705  (2743314..2744138) |
| **Cationic antimicrobial peptides resistance** | [K03367](https://www.kegg.jp/entry/K03367) | dltA;D-alanine--poly(phosphoribitol) ligase subunit 1 [EC:6.1.1.13] | fig\|1590.2604.peg.1709  (1753263..1754789) | fig\|1590.2594.peg.1356 (1368877..1370403) |  | fig\|1590.2644.peg.1293  (1292247..1293773) | fig\|1590.2648.peg.1275  (1307571..1309097) | fig\|1590.2646.peg.1391  (1397638..1399164) |
|  | [K03739](https://www.kegg.jp/entry/K03739) | dltB; membrane protein involved in D-alanine export | fig\|1590.2604.peg.1708  (1752052..1753266) | fig\|1590.2594.peg.1357 (1370400..1371614) |  | fig\|1590.2644.peg.1294 (1293770..1294984) | fig\|1590.2648.peg.1276  (1309094..1310308) | fig\|1590.2646.peg.1392  (1399161..1400375) |
|  | [K14188](https://www.kegg.jp/entry/K14188) | dltC;  D-alanine--poly(phosphoribitol) ligase subunit 2 [EC:6.1.1.13] | fig\|1590.2604.peg.1165  (1211981..1212217) | fig\|1590.2594.peg.1358  (1371644..1371880) |  | fig\|1590.2644.peg.1295 (1295014..1295250) | fig\|1590.2648.peg.1277  (1310338..1310574) | fig\|1590.2646.peg.1393  (1400405..1400641) |
|  | [K14188](https://www.kegg.jp/entry/K14188) | dltC;  D-alanine--poly(phosphoribitol) ligase subunit 2 [EC:6.1.1.13] | fig\|1590.2604.peg.1707  (1751786..1752022) | fig\|1590.2594.peg.1871  (1887308..1887544) |  | fig\|1590.2644.peg.1829 (1823820..1824056) | fig\|1590.2648.peg.1845  (1859289..1859525) | fig\|1590.2646.peg.1913  (1919299..1919535) |
|  | [K03740](https://www.kegg.jp/entry/K03740) | dltD; D-alanine transfer protein | fig\|1590.2604.peg.1706  (1750512..1751789) | fig\|1590.2594.peg.1359  (1371877..1373154) |  | fig\|1590.2644.peg.1296  (1295247..1296524) | fig\|1590.2648.peg.1278  (1310571..1311848) | fig\|1590.2646.peg.1394  (1400638..1401915) |
|  | [K14205](https://www.kegg.jp/entry/K14205) | mprF, fmtC; phosphatidylglycerol lysyltransferase [EC:2.3.2.3] | fig\|1590.2604.peg.576  (596412..599015) | fig\|1590.2594.peg.2553  (2582911..2585514) |  | fig\|1590.2644.peg.2391  (2413652..2416255) | fig\|1590.2648.peg.2504  (2545155..2547758) | fig\|1590.2646.peg.2528  (2563606..2566209) |
| **Macrolide resistance,** | [K18231](https://www.kegg.jp/entry/K18231) | msr, vmlR; macrolide transport system ATP-binding/permease protein | fig\|1590.2604.peg.253  (261638..263158) | fig\|1590.2594.peg.2967  (2994264..2995784) | fig\|1590.2643.peg.2636  (2672172..2673692) | fig\|1590.2644.peg.2724  (2763606..2765126) | fig\|1590.2648.peg.2841  (2899138..2900658) | fig\|1590.2646.peg.2924  (2960169..2961689) |
| **Multidrug resistance** | [K18907](https://www.kegg.jp/entry/K18907) | NorG; GntR family transcriptional regulator, regulator for abcA and norABC | fig\|1590.2604.peg.1166  (1212326..1213777) | fig\|1590.2594.peg.1870  (1885748..1887199) | fig\|1590.2643.peg.1603  (1612286..1613737) | fig\|1590.2644.peg.2391  (2413652..2416255) | fig\|1590.2648.peg.1844  (1857729..1859180) | fig\|1590.2646.peg.1912  (1917739..1919190) |
| **Multidrug resistance** | [K18104](https://www.kegg.jp/entry/K18104) | abcA, bmrA; ATP-binding cassette, subfamily B, bacterial AbcA/BmrA [EC:7.6.2.2] | fig\|1590.2604.peg.2127  (2152659..2154422)* | fig\|1590.2594.peg.545  (564429..566186) | fig\|1590.2643.peg.294 (299716..301473) | fig\|1590.2644.peg.490  (490450..492207) | fig\|1590.2648.peg.521  (542069..543826) | fig\|1590.2646.peg.523  (549113..550870) |
| **Multidrug resistance** | [K18104](https://www.kegg.jp/entry/K18104) | abcA, bmrA; ATP-binding cassette, subfamily B, bacterial AbcA/BmrA [EC:7.6.2.2] | fig\|1590.2604.peg.2562  (2583352..2584959) | fig\|1590.2594.peg.934  (965903..967666) | fig\|1590.2643.peg.679 (701265..703028) | fig\|1590.2644.peg.880  (893910..895673) | fig\|1590.2648.peg.906  (945657..947420) | fig\|1590.2646.peg.907 (950811..952574) |
| **Multidrug resistance** | [K18907](https://www.kegg.jp/entry/K18907) | norG; GntR family transcriptional regulator, regulator for abcA and norABC | fig\|1590.2604.peg.1166  (1212326..1213777) | fig\|1590.2594.peg.1870  (1885748..1887199) | fig\|1590.2643.peg.1603  (1612286..1613737) | fig\|1590.2644.peg.1828  (1822260..1823711) | fig\|1590.2648.peg.1844  (1857729..1859180) | fig\|1590.2646.peg.1912  (1917739..1919190) |
| **Multidrug resistance** | [K18908](https://www.kegg.jp/entry/K18908) | mepA; Multidrug resistance, efflux pump MepA | fig\|1590.2604.peg.142  (143795..145129) | fig\|1590.2594.peg.3079  (3110314..3111648) | fig\|1590.2643.peg.2748  (2788242..2789576) | fig\|1590.2644.peg.2834  (2879660..2880994) | fig\|1590.2648.peg.2953  (3015206..3016540) | fig\|1590.2646.peg.3036  (3076244..3077578) |
| **Tetracycline, resistance** | [K18220](https://www.kegg.jp/entry/K18220) | tetM, tetO; tetracycline, ribosomal protection resistance protein | fig\|1590.2604.peg.134  (137086..139104) | fig\|1590.2594.peg.3089  (3116318..3118336) | fig\|1590.2643.peg.2757  (2794253..2796271) | fig\|1590.2644.peg.2842  (2885665..2887683) | fig\|1590.2648.peg.2961  (3021208..3023226) | fig\|1590.2646.peg.3045  (3082249..3084267) |
| **Phenicol resistance** | [K19271](https://www.kegg.jp/entry/K19271) | catA; chloramphenicol O-acetyltransferase type A [EC:2.3.1.28] | fig\|1590.2604.peg.1476  (1517901..1518569) | fig\|1590.2594.peg.1551 (1568159..1568827 | fig\|1590.2643.peg.1219  (1250835..1251503 | fig\|1590.2644.peg.1501 (1502542..1503210 | fig\|1590.2648.peg.1468  (1506486..1507154) | fig\|1590.2646.peg.1585  (1597484..1598152) |
| **Vancomycin resistance** | [K07260](https://www.kegg.jp/entry/K07260) | vanY; zinc D-Ala-D-Ala carboxypeptidase [EC:3.4.17.14] | fig\|1590.2604.peg.862  (888643..889380) | fig\|1590.2594.peg.2257  (2279104..2279841) | fig\|1590.2643.peg.1933 (1965178..1965915) | fig\|1590.2644.peg.2101  (2116235..2116972) | fig\|1590.2648.peg.2218  (2246922..2247659) | fig\|1590.2646.peg.2232  (2260999..2261736) |
| **Vancomycin resistance** | [K08641](https://www.kegg.jp/entry/K08641) | vanX; zinc D-Ala-D-Ala dipeptidase [EC:3.4.13.22] | fig\|1590.2604.peg.632  (645980..646537) | fig\|1590.2594.peg.2469  (2501871..2502428) | fig\|1590.2643.peg.2145  (2186696..2187253) | fig\|1590.2644.peg.2315  (2339140..2339697) | fig\|1590.2648.peg.2427  (2470712..2471269) | fig\|1590.2646.peg.2445  (2483799..2484356) |

* The predicted feature located in an incomplete phage in *L. plantarum* PROBI S126

1. Kanehisa, M. & Goto, S. KEGG: Kyoto Encyclopedia of Genes and Genomes. Nucleic Acids Res. 28, 27–30 (2000).

2. Kanehisa, M. Toward understanding the origin and evolution of cellular organisms. Protein Sci. 28, 1947–1951 (2019).

3. Kanehisa, M., Furumichi, M., Sato, Y., Kawashima, M. & Ishiguro-Watanabe, M. KEGG for taxonomy-based analysis of pathways and genomes. Nucleic Acids Res. (2022) bbdoi:10.1093/NAR/GKAC963.

**Table S5. List of predicted prophages and their locations in the selected TanA+ *L. plantarum* strains.**

| **Strain** | **Region** | **Region Length** | **Phage completeness** | **# Total Proteins** | **Location** |
| --- | --- | --- | --- | --- | --- |
| **PROBI S126** | 1 | 17Kb | Questionable | 24 | 85329-102328 |
|  | 2 | 8.2Kb | Incomplete | 9 | 1032157-1040374 |
|  | 3 | 49.3Kb | Intact | 41 | 1548103-1597459 |
|  | 4 | 10.5Kb | Incomplete | 14 | 1799517-1810096 |
|  | 5 | 31.4Kb | Questionable | 30 | 2090593-2122075 |
|  | 6 | 18Kb | Incomplete | 33 | 2125306-2143364 |
|  | 7 | 43.3Kb | Intact | 55 | 2152659-2195966 |
| **PROBI S204** | 1 | 47.8Kb | Intact | 60 | 976535-1024416 |
|  | 2 | 40.3Kb | Intact | 52 | 1925653-1965990 |
|  | 3 | 39.9Kb | Intact | 56 | 2586491-2626413 |
| **RKG 1-473** | 1 | 49.7Kb | Intact | 56 | 1333404-1383122 |
|  | 2 | 7.2Kb | Questionable | 12 | 1785867-1793081 |
|  | 3 | 50.3Kb | Intact | 57 | 2329516-2379858 |
|  | 4 | 17.5Kb | Questionable | 23 | 2830200-2847702 |
| **RKG 1-500** | 1 | 53.3Kb | Intact | 61 | 904755-958074 |
|  | 2 | 33Kb | Questionable | 27 | 3031582-3064591 |
| **RKG 2-219** | 1 | 46.5Kb | Intact | 44 | 1587751-1634264 |
|  | 2 | 15.2Kb | Incomplete | 26 | 1621830-1637078 |
|  | 3 | 42.3Kb | Intact | 54 | 1895372-1937720 |
|  | 4 | 23.6Kb | Questionable | 12 | 2042982-2066636 |
|  | 5 | 12Kb | Incomplete | 16 | 2114413-2126418 |
| **RKG 2-690** | 1 | 49.2Kb | Intact | 62 | 961656-1010921 |
|  | 2 | 40.1Kb | Intact | 46 | 1150315-1190469 |
|  | 3 | 21.2Kb | Incomplete | 24 | 2605371-2626604 |
|  | 4 | 17.1Kb | Incomplete | 26 | 2627648-2644813 |

**Table S6. List of enzymes involved in biogenic amine production in the selected TanA+ *L. plantarum* strains.**

| **Polyamine biosynthesis features** | | | | ***L. plantarum strain*** | | | | | |
| --- | --- | --- | --- | --- | --- | --- | --- | --- | --- |
| **Amino acid pathway** | **KEGG_ID**^2,3,4^ | **Enzyme** | **Function** ^1^ | **PROBI  S126** | **PROBI  S204** | **RKG 1-473** | **RKG 1-500** | **RKG 2-219** | **RKG 2-690** |
| **Lysine degradation** | K01582 | Lysine decarboxylase [EC:4.1.1.18] | lysine → agmatine | Absent | Absent | Absent | Absent | Absent | Absent |
|  | K23385 | D-ornithine/D-lysine decarboxylase [EC:4.1.1.116] | arginine → agmatine | Absent | Absent | Absent | Absent | Absent | Absent |
| **Arginine and proline metabolism** | K01583 K01584 K01585 K02626 | Arginine decarboxylase [EC:4.1.1.19] | arginine → agmatine | Absent | Absent | Absent | Absent | Absent | Absent |
|  | K01480 | Agmatinase [EC:3.5.3.11] | agmatine → putrescine | Absent | Absent | Absent | Absent | Absent | Absent |
|  | K00797 | Spermidine synthase [EC:2.5.1.16] | putrescine → spermidine, spermine | fig\|1590.2604.peg.2285 | fig\|1590.2594.peg.601 | fig\|1590.2643.peg.347 | Absent | fig\|1590.2595.peg.802 | fig\|1590.2596.peg.577 |
|  | K01476 | Arginase [EC:3.5.3.1] | arginine → ornithine | Absent | Absent | Absent | Absent | Absent | Absent |
|  | K01581 | Ornithine decarboxylase [EC:4.1.1.17] | ornithine → putrescine | Absent | Absent | Absent | Absent | Absent | Absent |
| **Histidine metabolism** | K01590 | Histidine decarboxylase [EC:4.1.1.22] | histidine → histamine | Absent | Absent | Absent | Absent | Absent | Absent |
| **Tyrosine metabolism** | K22329 K22330 K01592 K18933 | Tyrosine decarboxylase [EC:4.1.1.25] | tyrosine → tyramine | Absent | Absent | Absent | Absent | Absent | Absent |
| **Tryptophan metabolism** | K01593 | Tryptophan decarboxylase [EC:4.1.1.28] | tryptophan → tryptamine | Absent | Absent | Absent | Absent | Absent | Absent |

1. Chokesajjawatee, N. et al. Safety Assessment of a Nham Starter Culture *Lactobacillus plantarum* BCC9546 via Whole-genome Analysis. Sci. Reports 2020 101 10, 1–12 (2020).

2. Kanehisa, M. & Goto, S. KEGG: Kyoto Encyclopedia of Genes and Genomes. Nucleic Acids Res. 28, 27–30 (2000).

3. Kanehisa, M. Toward understanding the origin and evolution of cellular organisms. Protein Sci. 28, 1947–1951 (2019).

4. Kanehisa, M., Furumichi, M., Sato, Y., Kawashima, M. & Ishiguro-Watanabe, M. KEGG for taxonomy-based analysis of pathways and genomes. Nucleic Acids Res. (2022) bbdoi:10.1093/NAR/GKAC963.

**Figure S7. Bacteriocin profile of *tanA* harboring strains.**

In light blue, the presence, and in gray, the absence of bacteriocin genes. Lanes 1 and 2 distinguish the two bacteriocin profiles within Tan+ strains. The phylogenetic tree was performed by the randomized axelerated maximum likelihood in the BV-BRC. Strains marked with an asterisk are predicted to have a functional TanA.

**a**

TanA multiple sequence alignment
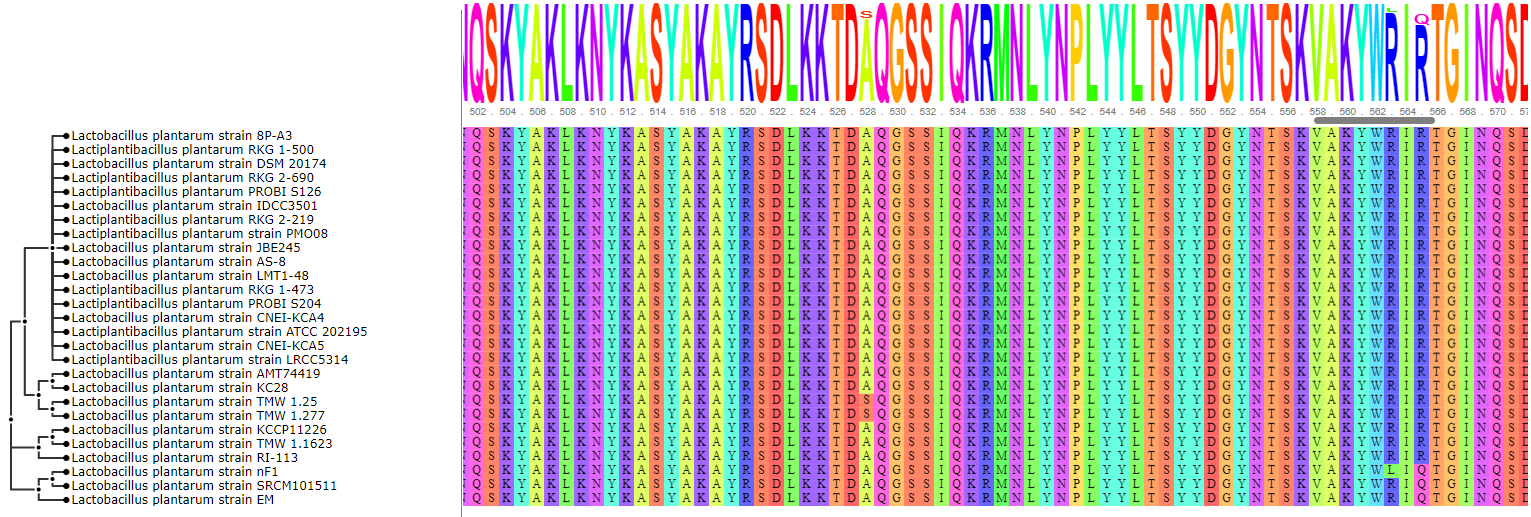


**b**

LpdB multiple sequence alignment


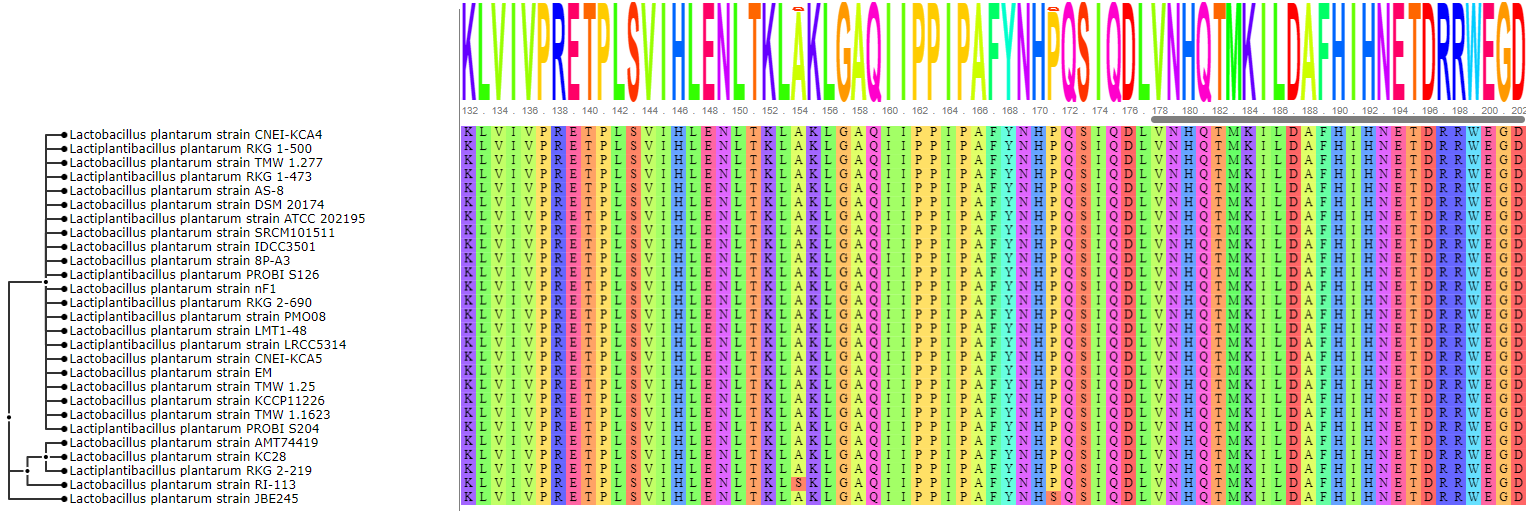


**c**

LpdC multiple sequence alignment
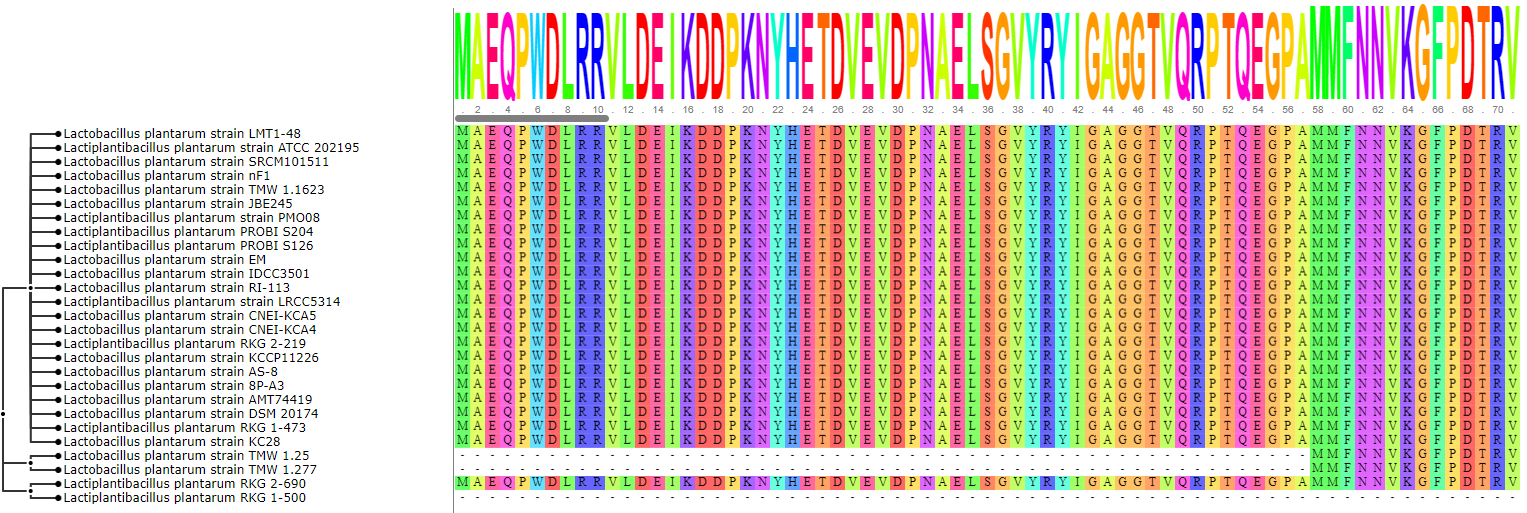


**Figure S8. Multiple sequence alignment of the genomic features encoding tannin-transforming enzymes in the**

***tanA-*harboring *L. plantarum* strains**

ATCC 14917 strain is referred as DSM 20174. RKG 1‑500 strain lacks gallate decarboxylase activity. Analysis performed in the BV-BRC

**Table S7. Sequences producing significant alignments with ATCC 14917 TanA protein**

| **Description** | **Scientific Name** | **Max Score** | **Total Score** | **Query Cover** | **E value** | **Per. ident** | **Acc. Len** | **Accession** |
| --- | --- | --- | --- | --- | --- | --- | --- | --- |
| tannase [*Lactiplantibacillus pentosus*] | *Lactiplantibacillus pentosus* | 1037 | 1037 | 100% | 0 | 90.91 | 627 | [WP_105920042.1](https://www.ncbi.nlm.nih.gov/protein/WP_105920042.1?report=genbank&log$=prottop&blast_rank=14&RID=B9F8XBWW016) |
| tannase [*Lactiplantibacillus pentosus*] | *Lactiplantibacillus pentosus* | 1036 | 1036 | 100% | 0 | 90.75 | 627 | [WP_245146520.1](https://www.ncbi.nlm.nih.gov/protein/WP_245146520.1?report=genbank&log$=prottop&blast_rank=16&RID=B9F8XBWW016) |
| tannase [*Lactiplantibacillus pentosus*] | *Lactiplantibacillus pentosus* | 1036 | 1036 | 100% | 0 | 90.59 | 627 | [WP_120768753.1](https://www.ncbi.nlm.nih.gov/protein/WP_120768753.1?report=genbank&log$=prottop&blast_rank=17&RID=B9F8XBWW016) |
| tannase [*Lactiplantibacillus pentosus*] | *Lactiplantibacillus pentosus* | 1035 | 1035 | 100% | 0 | 90.43 | 627 | [WP_159258175.1](https://www.ncbi.nlm.nih.gov/protein/WP_159258175.1?report=genbank&log$=prottop&blast_rank=18&RID=B9F8XBWW016) |
| tannase [*Lactiplantibacillus pentosus*] | *Lactiplantibacillus pentosus* | 1035 | 1035 | 100% | 0 | 90.59 | 627 | [WP_216777527.1](https://www.ncbi.nlm.nih.gov/protein/WP_216777527.1?report=genbank&log$=prottop&blast_rank=20&RID=B9F8XBWW016) |
| tannase [*Lactiplantibacillus pentosus*] | *Lactiplantibacillus pentosus* | 1035 | 1035 | 100% | 0 | 90.75 | 627 | [WP_101873413.1](https://www.ncbi.nlm.nih.gov/protein/WP_101873413.1?report=genbank&log$=prottop&blast_rank=21&RID=B9F8XBWW016) |
| tannase [*Lactiplantibacillus pentosus*] | *Lactiplantibacillus pentosus* | 1033 | 1033 | 100% | 0 | 90.59 | 627 | [WP_050338551.1](https://www.ncbi.nlm.nih.gov/protein/WP_050338551.1?report=genbank&log$=prottop&blast_rank=22&RID=B9F8XBWW016) |
| tannase [*Lactiplantibacillus pentosus*] | *Lactiplantibacillus pentosus* | 1033 | 1033 | 100% | 0 | 90.59 | 627 | [WP_105924271.1](https://www.ncbi.nlm.nih.gov/protein/WP_105924271.1?report=genbank&log$=prottop&blast_rank=23&RID=B9F8XBWW016) |
| tannase [*Lactiplantibacillus pentosus*] | *Lactiplantibacillus pentosus* | 1033 | 1033 | 100% | 0 | 90.59 | 627 | [WP_216779486.1](https://www.ncbi.nlm.nih.gov/protein/WP_216779486.1?report=genbank&log$=prottop&blast_rank=24&RID=B9F8XBWW016) |
| tannase [*Lactiplantibacillus pentosus*] | *Lactiplantibacillus pentosus* | 1033 | 1033 | 100% | 0 | 90.43 | 627 | [WP_226768928.1](https://www.ncbi.nlm.nih.gov/protein/WP_226768928.1?report=genbank&log$=prottop&blast_rank=26&RID=B9F8XBWW016) |
| tannase [*Lactiplantibacillus pentosus*] | *Lactiplantibacillus pentosus* | 1033 | 1033 | 100% | 0 | 90.59 | 627 | [WP_122210964.1](https://www.ncbi.nlm.nih.gov/protein/WP_122210964.1?report=genbank&log$=prottop&blast_rank=27&RID=B9F8XBWW016) |
| tannase [*Lactiplantibacillus pentosus*] | *Lactiplantibacillus pentosus* | 1032 | 1032 | 100% | 0 | 90.43 | 627 | [WP_216804345.1](https://www.ncbi.nlm.nih.gov/protein/WP_216804345.1?report=genbank&log$=prottop&blast_rank=28&RID=B9F8XBWW016) |
| tannase [*Lactiplantibacillus pentosus*] | *Lactiplantibacillus pentosus* | 1032 | 1032 | 100% | 0 | 90.43 | 627 | [WP_105962082.1](https://www.ncbi.nlm.nih.gov/protein/WP_105962082.1?report=genbank&log$=prottop&blast_rank=29&RID=B9F8XBWW016) |
| tannase [*Lactiplantibacillus pentosus*] | *Lactiplantibacillus pentosus* | 1031 | 1031 | 100% | 0 | 90.43 | 627 | [WP_122217882.1](https://www.ncbi.nlm.nih.gov/protein/WP_122217882.1?report=genbank&log$=prottop&blast_rank=30&RID=B9F8XBWW016) |
| tannase [*Lactiplantibacillus pentosus*] | *Lactiplantibacillus pentosus* | 1030 | 1030 | 100% | 0 | 90.27 | 627 | [WP_209042125.1](https://www.ncbi.nlm.nih.gov/protein/WP_209042125.1?report=genbank&log$=prottop&blast_rank=31&RID=B9F8XBWW016) |
| tannase [*Lactiplantibacillus pentosus*] | *Lactiplantibacillus pentosus* | 1030 | 1030 | 100% | 0 | 90.43 | 627 | [WP_105921976.1](https://www.ncbi.nlm.nih.gov/protein/WP_105921976.1?report=genbank&log$=prottop&blast_rank=32&RID=B9F8XBWW016) |
| hypothetical protein [*Levilactobacillus zymae*] | *Levilactobacillus zymae* | 1023 | 1023 | 99% | 0 | 85.44 | 620 | [WP_057733716.1](https://www.ncbi.nlm.nih.gov/protein/WP_057733716.1?report=genbank&log$=prottop&blast_rank=33&RID=B9F8XBWW016) |
| Esterase/lipase [*Levilactobacillus zymae*] | *Levilactobacillus zymae* | 1019 | 1019 | 99% | 0 | 85.28 | 620 | [SMS13414.1](https://www.ncbi.nlm.nih.gov/protein/SMS13414.1?report=genbank&log$=prottop&blast_rank=34&RID=B9F8XBWW016) |
| hypothetical protein [*Levilactobacillus spicheri*] | *Levilactobacillus spicheri* | 1019 | 1019 | 99% | 0 | 85.12 | 620 | [WP_045807561.1](https://www.ncbi.nlm.nih.gov/protein/WP_045807561.1?report=genbank&log$=prottop&blast_rank=35&RID=B9F8XBWW016) |
| tannase [*Lactiplantibacillus pentosus*] | *Lactiplantibacillus pentosus* | 1017 | 1017 | 100% | 0 | 89.47 | 627 | [WP_088771207.1](https://www.ncbi.nlm.nih.gov/protein/WP_088771207.1?report=genbank&log$=prottop&blast_rank=36&RID=B9F8XBWW016) |
| tannase [*Levilactobacillus zymae* DSM 19395] | *Levilactobacillus zymae* DSM 19395 | 1008 | 1008 | 98% | 0 | 85.88 | 611 | [KRL08696.1](https://www.ncbi.nlm.nih.gov/protein/KRL08696.1?report=genbank&log$=prottop&blast_rank=37&RID=B9F8XBWW016) |
| tannase [*Levilactobacillus zymae*] | *Levilactobacillus zymae* | 984 | 984 | 91% | 0 | 86.34 | 581 | [WP_231921676.1](https://www.ncbi.nlm.nih.gov/protein/WP_231921676.1?report=genbank&log$=prottop&blast_rank=38&RID=B9F8XBWW016) |
| tannase [*Levilactobacillus suantsaii*] | *Levilactobacillus suantsaii* | 928 | 928 | 96% | 0 | 81.35 | 604 | [WP_233449154.1](https://www.ncbi.nlm.nih.gov/protein/WP_233449154.1?report=genbank&log$=prottop&blast_rank=43&RID=B9F8XBWW016) |
| tannase [*Levilactobacillus suantsaii*] | *Levilactobacillus suantsaii* | 925 | 925 | 96% | 0 | 81.19 | 604 | [WP_225423562.1](https://www.ncbi.nlm.nih.gov/protein/WP_225423562.1?report=genbank&log$=prottop&blast_rank=44&RID=B9F8XBWW016) |

Analysis performed using NCBI’s BLAST and database.
